# The association of the planetary health diet with type 2 diabetes incidence and greenhouse gas emissions: Findings from the EPIC-Norfolk prospective cohort study

**DOI:** 10.1371/journal.pmed.1004633

**Published:** 2025-09-16

**Authors:** Solomon A. Sowah, Fumiaki Imamura, Daniel B. Ibsen, Pablo Monsivais, Nicholas J. Wareham, Nita G. Forouhi

**Affiliations:** 1 Medical Research Council Epidemiology Unit, Institute of Metabolic Science, School of Clinical Medicine, University of Cambridge, Cambridge, United Kingdom; 2 Steno Diabetes Center Aarhus, Aarhus University Hospital, Aarhus, Denmark,; 3 Department of Public Health, Aarhus University, Aarhus, Denmark; 4 Department of Nutrition, Sports and Exercise, University of Copenhagen, Frederiksberg, Denmark; 5 Department of Nutrition and Exercise Physiology, Elson S. Floyd College of Medicine, Washington State University, Spokane, Washington, United States of America; Waymark, UNITED STATES OF AMERICA

## Abstract

**Background:**

The planetary health diet (PHD) has been proposed as a dietary index with potential co-benefits for human and planetary health. Evidence is limited on its association with type 2 diabetes (T2D) incidence and greenhouse gas (GHG) emissions. Our objective was to assess the associations of adherence to the PHD with incident T2D and GHG emissions.

**Methods and findings:**

We analysed data from 23,722 participants (55% female), with a mean (standard deviation, SD) age of 59.1 (9.3) in the UK-based EPIC-Norfolk prospective cohort study. Dietary intake was assessed across three time points (1993–1997, 1998–2000 and 2004–2011) using a food frequency questionnaire. We assessed adherence to the PHD (theoretical score range 0–140 points) based on the consumption of 13 food groups and two nutrients. Cox proportional hazards regression models, which accounted for time-varying covariates, were used to estimate the hazard ratios (HRs) and 95% confidence intervals (CIs) for T2D. Linear regression models were used to analyse the association between the PHD and estimated GHG emissions.

During a mean follow-up period of 19.4 (SD 6.8) years, 3,496 cases of incident T2D were recorded over 461,086 person-years. Greater adherence to the PHD was associated with lower T2D incidence; comparing the highest PHD quintile (85.7–117.8 points) to the lowest (33.9–68.4 points), the HR (95% CI) was 0.68 (0.61, 0.76) in the most adjusted model including socio-demographic, behavioural factors, energy intake, adiposity, and prevalent cardiovascular disease or cancer. The estimated population attributable fraction (PAF) for incident T2D due to adherence below the 80th percentile (85.7 points) was 12.3% (95% CI: 9.2%, 15.3%). Those in the highest quintile of the PHD had approximately 18% lower GHG emissions compared to those in the lowest (*β*_5th/1st_ −18.4% (95% CI: −19.3%, −17.5%)). The main limitation of this research is the possibility of residual confounding due to the observational design of this study.

**Conclusions:**

Our findings of a lower incidence of T2D and reduced GHG emissions among those with higher adherence to the PHD support the promotion of this diet for the population-level prevention of T2D and for planetary sustainability.

## Author summary

Why was this study done?

- There are growing concerns about the adverse effects of unhealthy dietary intake on both human and planetary health.
- In 2019, the EAT-Lancet Commission on Food, Planet, Health recommended adherence to a newly developed planetary health diet (PHD) to promote human health and environmental sustainability.
- There is limited epidemiological evidence on the association of adherence to the PHD with type 2 diabetes (T2D) incidence and with adverse environmental impact indicators such as greenhouse gas (GHG) emissions. Furthermore, evidence on the association between changes in adherence to the PHD and subsequent T2D incidence is sparse.

What did the researchers do and find?

- We assessed adherence to the PHD among 23,722 adults in the UK and we estimated the association of adherence to the PHD with T2D incidence and diet-related GHG emissions. Additionally, we evaluated the association between changes in adherence to the PHD and subsequent T2D incidence.
- During an average follow-up over approximately 20 years, 3,496 new cases of T2D were recorded. Higher adherence to the PHD was associated with lower T2D incidence and lower GHG emissions.
- T2D incidence was higher among those who reported a decrease in adherence to the PHD of more than 10% during the follow-up period compared to those who maintained stable adherence, defined as a change in adherence to the PHD of less than 3%, either as a decrease or increase.

What do these findings mean?

- Maintaining high adherence to the PHD is important for reducing the risk of developing T2D, although more evidence is needed on how different patterns of changes in adherence to the PHD over the long term may influence the risk of T2D.
- Promoting adherence to the PHD in the general population should be considered as part of strategies aimed at the prevention of T2D and also for simultaneously reducing the adverse environmental impact linked to unhealthy dietary intake.
- The current observational study design limits the inference of a causal link between the PHD and T2D since residual confounding from unknown or unmeasured factors or factors assessed with measurement error cannot be ruled out.

## Abstract 3

Using data from the EPIC-Norfolk study, Solomon A. Sowah and colleagues assess the association between adherence to the Planetary Health Diet and incidence of type 2 diabetes and greenhouse gas emissions.

## Introduction

Type 2 diabetes (T2D) accounts for a substantial proportion of the global disease burden, and its rising prevalence has been fuelled by increasing obesity, predominantly due to unhealthy diets, among other modifiable factors such as physical inactivity [1]. Accumulating research evidence suggests that the habitual consumption of unhealthy diets negatively impacts both human health and the environment [2]. In 2019, the EAT-Lancet Commission on Healthy Diets from Sustainable Food Systems developed dietary recommendations aimed at reducing the risk of noncommunicable diseases as well as the negative environmental impacts of unhealthy food consumption [3]. The recommendations emphasised the consumption of higher amounts of healthful plant foods such as whole grains, fruits, vegetables and legumes, and lower amounts of less healthful plant foods, such as sugar-sweetened beverages, and animal-derived foods, particularly red and processed meat [3]. However, epidemiological evidence about the association between the EAT-Lancet reference diet—the so-called planetary health diet (PHD)—and the risk of T2D and diet-related GHG emissions is limited and inconsistent [4–9].

Previous inconsistent findings may be due to variations in the characteristics of the cohorts analysed, and the criteria used to assess adherence to the PHD, often based on a dichotomous assessment of adherence to each food component of the PHD [4,6–8]. Other factors may include random and systematic temporal variations in dietary intakes that were not captured in most of the longitudinal cohort studies without repeated dietary assessment [10]. Moreover, no study has evaluated whether changes in adherence to the PHD in adulthood might influence subsequent risk of T2D. Thus, the association between the PHD and the risk of T2D may not have been adequately characterised and quantified. Also, while it has been reported that population-wide adoption of the PHD could prevent approximately 19% of premature deaths by 2030 [3]; similar population impact estimates for T2D are sparse [5].

Our primary aim was to assess the association of the PHD with T2D incidence and GHG emissions in a population-based cohort in the United Kingdom, and to estimate the potential reduction in T2D cases if all participants had maintained the high level of adherence to the PHD. Our secondary objectives included the assessment of the association between changes in adherence to the PHD and the subsequent risk of T2D. We additionally characterised the associations of the PHD with cardiometabolic risk markers and selected nutritional biomarkers typically associated with high consumption of plant foods, and assessed whether these factors may influence the PHD-T2D association.

## Methods

### Study design and participants

We analysed data from the EPIC-Norfolk study, a prospective cohort study in the UK. The details of the design of the EPIC-Norfolk study have previously been described elsewhere [11]. Briefly, the study recruited 25,639 adults (55% female) aged 40–79 years through General Practices in Norfolk, England, from 1993 to 1997. Participants underwent three assessments: a baseline assessment from 1993 to 1997, a first follow-up assessment from 1998 to 2000 (*n* = 15,786), and a second follow-up assessment from 2004 to 2011 (*n* = 8,623).

For the present analysis, we excluded participants with prevalent T2D (*n* = 697), those with no data on dietary intake at baseline (*n* = 890), and those with missing baseline data for any of the covariates to adjust for in the most adjusted primary analysis (*n* = 330). Therefore, the sample size for this current study was 23,722 (S1 Fig). The EPIC-Norfolk study was approved by the Norwich District Health Authority Ethics Committee (Ref. 98NC01), and all participants provided written informed consent. This study is reported as per the Strengthening the Reporting of Observational Studies in Epidemiology (STROBE) guideline (S1 Checklist).

### Dietary assessment and calculation of planetary health diet score

Dietary intake was assessed using a 130-item semi-quantitative food frequency questionnaire (FFQ) at baseline and during the follow-up phases. Validity of the FFQ was previously assessed against 24-h recalls, 16-day weighed-dietary records, and selected nutritional biomarkers in a subsample of participants in EPIC-Norfolk [12,13]. To reduce potential within-individual variation in dietary intake estimates over time, dietary intake at each follow-up was calculated as the cumulative average of dietary intake data collected up to that time, whenever possible [10]. FFQs obtained after diagnosis of major noncommunicable diseases, i.e., cardiovascular disease (CVD) or cancer, were not included when calculating the cumulative average because such a diagnosis may result in changes to dietary intake [10].

Adherence to the PHD was assessed using a previously described method as documented S1 Table [14,15]. For components for which the PHD recommends higher consumption, such as whole grains, fruits, vegetables and unsaturated fats, a minimum score of zero was assigned to non-consumption, and a maximum score of 10 was awarded if the amount of consumption was equal to or above the recommended target amount. Intakes greater than 0 g/d, but below the recommended target amounts were scored proportionally. For the total legumes component of the PHD, intake of non-soy legumes and soy legumes (soybean) were scored separately, and thus each score was weighted by a factor of 0.5. The components for which moderate intakes were recommended include red and processed meat, poultry, saturated and *trans-*fatty acids, dairy, added sugars and fruit juice. For each of those components, reverse scoring was assigned: a score of 10 was awarded for  consumption equal to or below a specified amount, a score of zero was awarded for consumption equal to or above a specified amount, and intakes outside of the specified amounts were scored proportionally. To obtain the overall score for adherence to the PHD, the scores were summed up to yield a score that ranged from zero (lowest) to 140 (highest) (S1 Table).

### Ascertainment of type 2 diabetes

Incident cases of T2D that occurred until 31 March 2020 were ascertained from a combination of multiple sources. These included self-report of a physician’s diagnosis of diabetes or self-reported diabetes medication usage on health and lifestyle follow-up questionnaires, or diabetes-specific medications brought to the first and second follow-up visits. Additional sources of case ascertainment included HbA1c measurements performed during the follow-up of the EPIC-Norfolk study, external sources curated by the National Health Service in England, such as hospital admission records from Hospital Episode Statistics, mortality data via record linkage to the UK Office of National Statistics with coding for diabetes, data on participants from the Diabetic Eye Screening Programme, and data from the National Diabetes Audit. Participants who self-reported a history of diabetes that could not be confirmed with any other sources of ascertainment were not considered as confirmed cases of T2D.

### Assessment of covariates, cardiometabolic risk markers and nutritional biomarkers

Participants’ demographic, health behaviour, and health information were obtained using a self-administered health and lifestyle questionnaire at baseline and also during two follow-up assessment visits. The level of physical activity was assessed by categorising it into a validated four-point index, i.e., inactive, moderately inactive, moderately active, and active, based on combined self-reported occupational and recreational physical activity levels [16]. During each assessment visit, height, weight, waist circumference (WC), and systolic and diastolic blood pressure (SBP and DBP) were measured by trained study nurses, and participants provided non-fasting plasma samples. Body mass index (BMI, kg/m^2^) was calculated as the weight (kg) divided by the squared height (m^2^). A detailed description of the assessments and samples collected at follow-up was previously published [11]. Further information on the methods used to assess additional cardiometabolic risk factors have been described in S1 Text. These include plasma glucose, HbA1c, C-reactive protein (CRP), alanine aminotransferase (ALT), aspartate aminotransferase (AST), gamma-glutamyl transferase (GGT), triglycerides, total cholesterol, low-density lipoprotein cholesterol (LDL-cholesterol), high-density lipoprotein cholesterol (HDL-cholesterol), and total-to-HDL cholesterol ratio. Details of the measurement of plasma vitamin C, carotenoids, alpha-tocopherol and gamma-tocopherol in the EPIC-Norfolk study have been published previously [17,18], and are described in S2 Text.

### Diet-related greenhouse gas emissions

The calculation of the GHG emissions for each of the 290 food codes in the food and nutrient database of the EPIC-Norfolk FFQ has previously been described [19]. The estimates were based on a life cycle assessment of the food commodities consumed in the UK from the earliest stages of production up to the retail distribution centres [19]. Individual-level GHG emissions were then derived as kg of CO_2_ equivalents per day (kgCO_2_eq/d). In the present study, GHG emissions data were available at baseline and the first follow-up.

### Statistical analyses

Participants were grouped into five groups based on the quintiles of their PHD scores at baseline. Descriptive statistics were obtained across the categories.

Cox proportional hazards regression models, with age as the underlying timescale, were used to calculate hazard ratios (HRs) and 95% confidence intervals (CIs) for the development of incident T2D. Follow-up time was calculated from the date of enrolment until the date of diagnosis of T2D, date of death, date of the start of a subsequent follow-up period, or date of administrative censoring (31 March 2020), whichever occurred first. The models fitted the repeated measures of PHD scores as continuous and categorical variables separately, and covariates were treated as time-varying covariates in the models if they varied over time and repeated measures were available. When follow-up covariate data were missing, the measurement from the preceding assessment period was used. Four models were fitted: Model 1 adjusted for age and sex; Model 2 adjusted for the covariates in Model 1, plus education level (primary/none: education until the age of 11 years; O-level: education until the age of 16 years; A-level: education until the age of 18 years; or bachelor’s degree or above), physical activity level (inactive, moderately inactive, moderately active, or active), smoking habits (never, former, or current), marital status (single, married, widowed, separated, or divorced), use of vitamin supplement (yes/no), family history of T2D (yes/no), alcohol consumption (g/d), and energy intake (kcal/d); Model 3 adjusted for covariates in Model 2, plus BMI (kg/m^2^). Model 4 further adjusted for prevalent CVD or cancer and was considered as the main model. We additionally fitted restricted-cubic splines based on the most adjusted model and evaluated the potential non-linearity of the PHD-T2D association by comparing two nested models: one with the spline terms and another without the spline terms, using a likelihood ratio test. The proportional hazards assumption was not violated by the PHD variable, based on an evaluation of the Schoënfeld residuals.

#### Population attributable fraction.

We calculated the population attributable fraction (PAF) to estimate the proportion of incident T2D cases that could have been prevented had all participants attained a score equal to the 80th percentile in this study population (85.7 points), assuming a causal association between the PHD and incidence of T2D. We used the formula *(Io − Ii)/Io* [20]: *Io* as the observed incidence of T2D in the study population; and *Ii* as the hypothetical ideal incidence if all participants had achieved the 80th percentile PHD score. Bootstrapping with 1,000 iterations was used to estimate the 95% CI for the PAF.

#### Effect modification, subgroup, and sensitivity analyses.

We assessed the interaction between the PHD and age, sex, BMI, and a family history of diabetes by testing the significance of cross-product terms of the PHD and each of these covariates using a likelihood ratio test. The associations of the PHD with T2D incidence were further evaluated across strata of age (<60 or >60 years), sex (male or female), BMI (<25, 25 to <30, or >30 kg/m^2^), and family history of diabetes (yes or no), adjusting for all covariates included in the most adjusted model. To evaluate the robustness of our primary results we performed several sensitivity analyses, as described in S3 Text. These analyses assessed how possible reverse causation, individual PHD components, potential measurement errors in dietary intake, missing data (using 10 imputed datasets), cardiometabolic risk markers, and hormone replacement therapy use in females influenced the primary results.

#### Secondary analyses.

We analysed the association between changes in adherence to the PHD and subsequent incidence of T2D by restricting the analyses to participants with dietary data at baseline and at least one follow-up assessment (*n* = 11,888; *n* cases = 1,299; person-years = 198,333). Because dietary data were not updated upon chronic disease diagnosis, only those with repeated dietary data who did not develop T2D or developed T2D only after providing dietary data at follow-up were included in this analysis. The changes were calculated as relative percentage changes. Modelling the changes in PHD score continuously and categorically separately, we used multivariable-adjusted Cox models, with age as the underlying timescale to calculate HRs and 95% CIs for incident T2D. As in the primary analyses, covariates were treated as time-varying covariates: we adjusted for the baseline PHD score and baseline covariate characteristics, and where available, changes in baseline covariates in parallel to the PHD changes evaluated. Details of further secondary analyses are presented in S4 Text.

The association between the PHD and estimated GHG emissions was evaluated using linear mixed models with a random intercept for those who provided repeated dietary measures, adjusting for age, sex, alcohol intake (g/d), and energy intake (kcal/d). The PHD score was modelled as quintiles and separately as a continuous variable. To evaluate the influence on the individual components of the PHD on this association, the models were refitted after iteratively excluding each component of the PHD.

## Results

### Characteristics of participants

This study included 23,722 participants (55% female), with a mean (SD) age of 59.1 (9.3) years, and a mean BMI of 26.3 (3.9) kg/m^2^. The PHD score in the cohort ranged from 33.9 to 118.0 points, and the mean GHG emission was 6.3 (2.4) kgCO_2_eq/d. The mean age and BMI at baseline were comparable across categories of the PHD score (Table 1). Participants who had high adherence to the PHD were more likely to be female, to have a university degree, to be physically active, and to have never smoked. Also, those with higher adherence to the PHD had lower GHG emissions.

**Table 1:** Characteristics of participants at baseline according to categories of adherence to the planetary health diet (PHD) score: EPIC-Norfolk study^1^.

	Q1 (*n* = 4,745)	Q2 (*n* = 4,745)	Q3 (*n* = 4,742)	Q4 (*n* = 4,745)	Q5 (*n* = 4,745)	Total (*n* = 23,722)
PHD score	62.5 (4.9)	71.7 (1.8)	77.3 (1.5)	82.6 (1.7)	91.5 (5.1)	77.1 (10.4)
PHD score, range	33.9–68.4	68.4–74.7	74.7–79.8	79.8–85.7	85.7–117.8	33.9–117.8
GHG emissions, kgCO_2_eq/d^*^	6.5 (1.0, 34.9)	6.2 (1.2, 48.5)	6.0 (0.6, 28.1)	5.8 (1.4, 23.0)	5.0 (1.0, 17.3)	5.8 (0.6, 48.5)
Age, years	58.4 (9.3)	59.1 (9.3)	59.3 (9.3)	59.6 (9.2)	58.8 (9.3)	59.1 (9.3)
BMI, kg/m^2^	26.5 (3.9)	26.5 (3.9)	26.4 (3.8)	26.2 (3.8)	25.8 (3.9)	26.3 (3.9)
Females, %	39.7	52.2	55.6	61.4	66.4	55.1
Physical activity level, %						
Inactive	34.2	31.5	29.0	28.5	25.1	29.7
Moderately inactive	25.2	28.9	30.7	29.8	29.9	28.9
Moderately active	22.8	21.5	22.9	23.0	24.6	23.0
Active	17.8	18.1	17.5	18.7	20.4	18.5
Education level, %						
None	40.7	38.9	36.6	34.2	30.2	36.1
O-level	10.1	10.7	10.9	10.0	10.3	10.4
A-level	40.7	39.9	39.2	41.4	41.7	40.6
Degree	8.6	10.5	13.3	14.4	17.8	12.9
Smoking habits, %						
Current	20.9	13.0	9.6	8.3	6.4	11.7
Former	41.7	41.7	41.8	41.2	42.7	41.8
Never	37.3	45.3	48.7	50.4	50.9	46.5
Alcohol intake, g/d	9.7 (15.3)	8.5 (13.0)	8.6 (12.7)	8.2 (11.6)	8.4 (11.6)	8.7 (12.9)
Family history of diabetes, %	11.9	12.5	12.5	13.0	12.5	12.5
Marital status, % married	82.9	83.0	83.6	81.1	78.1	81.8
Use of vitamin supplement, % users	30.2	38.6	41.8	47.2	52.5	42.0
Dietary intakes, g/d or kcal/d						
Total energy	2,065 (614)	2,062 (606)	2,072 (605)	2,051 (584)	2,001 (591)	2,050 (601)
Wholegrain	37.0 (53.4)	71.1 (71.8)	100.8 (87.7)	120.6 (88.4)	147.8 (94.9)	95.5 (89.3)
Starchy vegetables	144.7 (80.3)	132.9 (68.0)	125.1 (64.1)	116.5 (60.2)	98.2 (56.7)	123.5 (68.2)
Vegetables^†^	132.0 (71.1)	166.6 (83.6)	187.9 (88.6)	211.5 (99.7)	249.0 (121.0)	189.4 (102.3)
Fruits	140.3 (137.1)	214.9 (165.7)	252.3 (164.9)	286.3 (178.9)	337.3 (214.5)	246.2 (186.3)
Dairy	456.9 (185.1)	435.5 (180.1)	429.3 (176.1)	413.7 (169.7)	381.8 (174.5)	423.4 (178.9)
Red meat or processed meat	76.1 (40.0)	69.6 (40.5)	63.6 (38.4)	55.5 (35.1)	38.3 (33.8)	60.6 (39.8)
Poultry	28.0 (22.6)	27.2 (19.9)	27.1 (20.8)	26.2 (20.8)	22.1 (22.2)	26.1 (21.4)
Egg	15.7 (14.3)	13.2 (13.0)	11.7 (11.1)	10.2 (9.6)	8.2 (8.8)	11.8 (11.8)
Fish and seafood	28.2 (22.9)	34.1 (22.7)	38.0 (23.5)	41.8 (26.3)	46.6 (30.2)	37.7 (26.0)
Nuts	1.6 (3.7)	2.0 (4.1)	2.6 (6.8)	3.1 (7.4)	6.7 (15.6)	3.2 (8.8)
Non-soy legumes	50.1 (32.2)	54.2 (32.3)	57.0 (35.4)	60.7 (36.2)	67.3 (41.5)	57.9 (36.2)
Soy legumes	0.2 (1.9)	0.4 (2.9)	0.6 (4.7)	1.1 (5.6)	4.5 (12.4)	1.3 (6.8)
Unsaturated fatty acids	33.5 (12.8)	34.2 (13.0)	34.7 (13.7)	35.0 (13.6)	35.8 (15.8)	34.6 (13.9)
Saturated and *trans-*fatty acids	36.5 (15.2)	34.1 (14.3)	32.6 (13.9)	30.8 (13.1)	27.7 (12.8)	32.4 (14.2)
Added sugar and fruit juice	84.2 (82.7)	77.2 (72.9)	70.4 (73.0)	57.0 (64.8)	42.8 (59.4)	66.3 (72.5)

^1^ Values are means (SD) for continuous variables (except the PHD score and GHG emissions), and percentages for categorical variables. Quintile 1 (Q1) represents the lowest adherence to the PHD, while Quintile 5 (Q5) represents the highest adherence. The total possible PHD score ranges from 0 (lowest) to 140 (highest).

* Diet-related GHG emissions data are presented as median (range).

† Total non-starchy vegetables. For GHG emissions data were missing for 37 participants.

BMI, body mass index; GHG, greenhouse gas; PHD score, planetary health diet score.

### PHD and T2D risk

**Fig 1:**
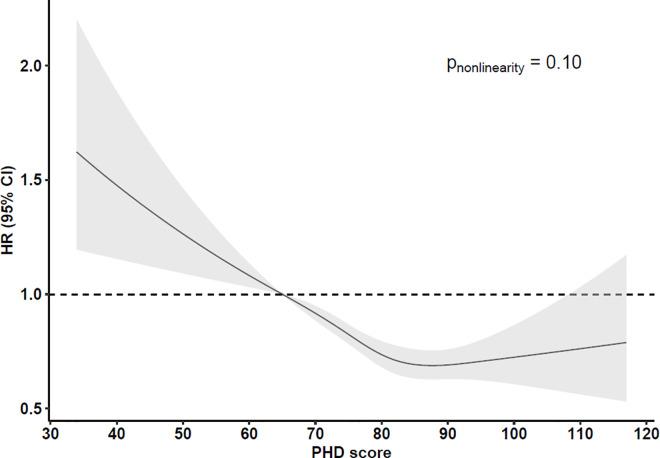
Association of the planetary health diet (PHD) score with incidence of type 2 diabetes. The hazard ratios (HR, solid line) and 95% confidence intervals (CI, grey) were estimated with Cox regression with a restricted cubic spline using four knots (5th, 35th, 65th and 95th percentiles). The 10th percentile of the PHD score (= 64.5 points) was used as a reference value.

During a mean follow-up of 19.4 (6.8) years, 3,496 cases of incident T2D were recorded. Higher adherence to the PHD was strongly inversely associated with T2D incidence. The HR (95% CI) for T2D comparing the highest quintile of the PHD score to the lowest was 0.68 (0.61, 0.76; *p* < 0.0001) in the most adjusted model (Table 2); and HR (95% CI) per 10-point higher score was 0.86 (0.83, 0.89), with no evidence of non-linearity (p for non-linearity = 0.10) (Fig 1). We estimated a PAF of 12.3% (95% CI: 9.2%, 15.3%) for incident T2D in the modelled scenario that all participants would have reported a PHD score equal to the 80th percentile (85.7 points).

**Table 2:** Association between the planetary health diet score and incident type 2 diabetes: EPIC-Norfolk study, the United Kingdom (*n* = 23,722)^1^^.^

	Q1	Q2	Q3	Q4	Q5		Per 10 points
n	4,745	4,745	4,742	4,745	4,745		
PHD score, range	33.9 - 68.4	68.4 - 74.7	74.7 - 79.8	79.8 - 85.67	85.7 - 117.8		
n cases (T2D)/person-years	918/88,517	755/90,344	670/92,708	593/93,728	560/95,788		3,496/461,086
n cases per-100,000 person-years	1,037	836	723	633	585		
adj. for age and sex^2^	1	0.84 (0.76, 0.92)	0.70 (0.64, 0.78)	0.63 (0.56, 0.70)	0.59 (0.53, 0.66)		0.82 (0.79, 0.85)
+ SES/ behaviours^3^	1	0.86 (0.78, 0.94)	0.74 (0.67, 0.82)	0.66 (0.60, 0.74)	0.64 (0.57, 0.71)		0.84 (0.82, 0.88)
+ BMI^4^	1	0.85 (0.77, 0.93)	0.74 (0.67, 0.81)	0.68 (0.61, 0.76)	0.68 (0.61, 0.76)		0.86 (0.83, 0.89)
+ prevalent CVD, cancer^5^	1	0.85 (0.77, 0.94)	0.74 (0.67, 0.81)	0.68 (0.61, 0.76)	0.68 (0.61, 0.76)		0.86 (0.83, 0.89)

^1^ Data are HR (95% CI). In the continuous analyses, the HRs and 95% CIs were estimated per 10-point higher score of the PHD score. Quintile 1 (Q1) represents the lowest adherence to the PHD, while Quintile 5 (Q5) represents the highest adherence. The total possible PHD score ranges from 0 (lowest) to 140 (highest).

^2^ Model 1: adjusted for age (years) and sex (male or female).

^3^ Model 2: adjusted for factors in model 1 plus physical activity (inactive, moderately inactive, moderately active, or active), smoking status (never, former, or current), level of education (none, O-level, A-level, or degree), use of vitamin supplements (yes/no), family history of diabetes (yes/no), alcohol intake (g/d) and energy intake (continuous, kcal/d).

^4^ Model 3: adjusted for factors in Model 2 plus body mass index (kg/m^2^).

^5^ Model 4: adjusted for factors in Model 3 plus prevalent CVD or cancer.

BMI, body mass index; CVD, cardiovascular disease; PHD, planetary health diet; SES, socioeconomic status; T2D, type 2 diabetes.

There was no evidence of effect modification of the association between the PHD and T2D incidence by age, sex, BMI, and family history of diabetes (*p*-interaction > 0.05) (S2 Table).

#### Sensitivity and secondary analyses.

The inverse association between the PHD and T2D incidence remained materially unchanged under a range of conditions when we: excluded participants who developed T2D within the first two years of follow-up or those who had HbA1c >6.5% at baseline; excluded those with prevalent CVD or cancer; excluded participants who reported implausibly high or low energy intake; additionally adjusted for use of HRT in females; additionally adjusted for diet quality using the Mediterranean diet score; or calculated the PHD score using baseline only FFQ data (S3 Table). The estimates were attenuated but remained significant when we additionally adjusted for cardiometabolic markers (HR comparing extreme quintiles: 0.75 (0.64, 0.88) or recalculated the PHD score by dichotomously scoring adherence to each component of the PHD (HR 0.84 [95% CI: 0.71, 0.98]) (S3 Table). The primary result also persisted when each component of the PHD was iteratively omitted from the calculation of the PHD score and simultaneously adjusted for in the Cox models (S2 Fig).

Furthermore, the results based on 10 imputed datasets were consistent with those from our primary analysis. Using the imputed datasets, the HRs (95% CIs) for T2D across quintiles two, three, four, and five of PHD adherence were, respectively, 0.85 (0.77, 0.94), 0.74 (0.66, 0.82), 0.68 (0.61, 0.76), and 0.68 (0.61, 0.76).

#### Changes in PHD and T2D incidence.

**Fig 2:**
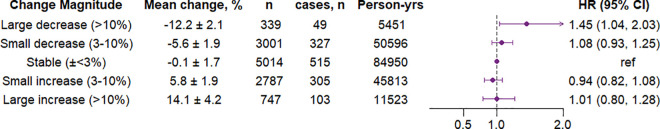
HR (95% CI) for type 2 diabetes according to the magnitude of change in planetary health diet score during follow-up. *n* = 11,888. The mean ± SD follow-up time from baseline to first follow-up was 10.9 ± 8.9 years; from first follow-up to second follow-up was 14.2 ± 5.6 years; and from second follow-up to end of follow-up was 10.3 ± 2.6 years. The mean ± SD PHD score at baseline, first follow-up and second follow-up was: 77.1 ± 10.4, 78.0 ± 9.2, and 79.7 ± 8.7, respectively. The change categories were constructed at both the first and second follow-ups as follows: an increase or decrease less than 3% was classified as ‘Stable’, a decrease greater than 10% was classified as ‘Large decrease’, a decrease from 3% to 10% was classified as ‘Small decrease’; an increase from 3% to 10% was classified as ‘Small increase’, and an increase greater than 10% classified as ‘Large increase’. ref = reference category. Estimates were derived from a Cox model that adjusted for the PHD score at baseline and for age (years), sex (male or female), physical activity (inactive, moderately inactive, moderately active, or active), energy intake (kcal/d), smoking status (never, former, or current), level of education (primary/none, O-level, A-level, or degree), use of multivitamin supplements (yes/no), family history of diabetes (yes/no), alcohol intake (g/d), BMI (kg/m^2^), and prevalent CVD or cancer, all at baseline, and also changes in physical activity level, smoking habits, energy intake, alcohol consumption, and BMI. BMI, body mass index; CI, confidence interval; CVD, cardiovascular disease; HR, hazard ratio; PHD, planetary health diet.

Mean (SD) change in the PHD per year was 0.03 (1.4) points. In the subset with available data (*n* = 11,888; *n* cases = 1,299; person-years = 198,333), there was no evidence of an association between changes in the PHD score and subsequent incident T2D with a HR per one point increase per year of 0.95 (95% CI: 0.71, 1.25). In the categorical analysis, participants who achieved a small increase (3%–10%) and a large increase in their PHD score (>10%) showed HRs (95% CIs) of 0.94 (0.82, 1.08) and 1.01 (0.80, 1.28) respectively, compared to those who maintained a relatively stable PHD score (±<3%). Those who decreased their PHD score by >10% showed a HR of 1.45 (95% CI: 1.04, 2.03) compared to those who maintained a stable PHD score (Fig 2).

#### The effect of nutritional biomarkers in the association of the PHD with T2D risk.

Plasma vitamin C and all six carotenoids were found to partly explain the inverse association between the PHD and incidence of T2D. There was some variation in the extent to which each individual biomarker explained the PHD-T2D association, ranging from 6.2% (cryptoxanthin) to 18.4% (beta-carotene) (S4 Table). Collectively, these biomarkers explained nearly a third of the association between the PHD and incidence of T2D (proportion explained = 31.6% [95% CI: 16.9%, 61.0%]).

### PHD and cardiometabolic risk markers

The PHD score was inversely associated with a range of cardiometabolic risk markers, including BMI, WC, plasma glucose, HbA1c, inflammatory marker CRP, and hepatic markers ALT and GGT, systolic and diastolic blood pressure, triglycerides, total cholesterol, and total-to-HDL cholesterol ratio (S3 Fig). A positive association was observed between the PHD score and HDL-cholesterol. There was no association between the PHD score and either AST or LDL-cholesterol.

### PHD and GHG emissions

**Fig 3:**
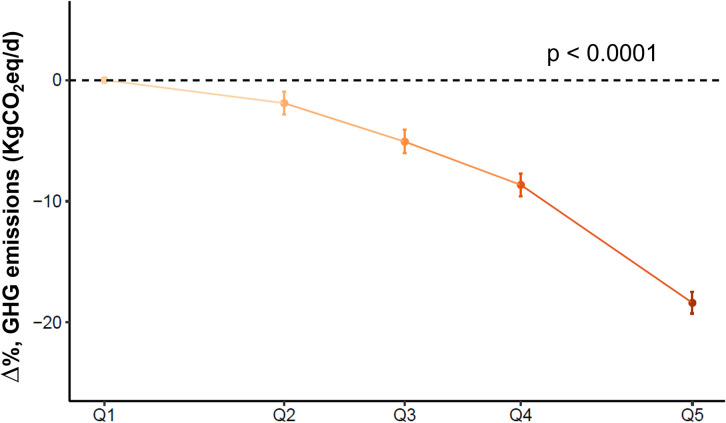
Association between the planetary health diet score and diet-related greenhouse gas (GHG) emissions (kgCO2eq/d). *n* = 23,685. Estimates were obtained in a linear regression analysis that adjusted for age (years), sex, energy intake (kcal/d), and alcohol intake (g/d). The estimates represent the expected percentage differences (95% CI) in the GHG emissions (kgCO_2_eq/d) for participants in PHD score quintiles 2, 3, 4, and 5 compared to those in quintile 1 (reference category). The quintiles on the x-axis are positioned at the median PHD scores for each group: 63.7, 71.8, 77.3, 82.5, and 90.2 for quintiles 1, 2, 3, 4, and 5, respectively. GHG, greenhouse gas, PHD, planetary health diet, Q1, Q2, Q3, Q4, and Q5, quintiles 1, 2, 3, 4, and 5, respectively.

The PHD score was inversely associated with diet-related GHG emissions in linear regression analysis, accounting for age, sex, alcohol intake, and energy intake. GHG emissions were consistently lower across quintiles of adherence to the PHD (*p* < 0.0001). Among those in the highest quintile, GHG emission was approximately 18% lower, compared to those in the lowest (*β*_5th/1st_ −18.4% (95% CI: −19.3%, −17.5%)) (Fig 3). A 10-point increment in the PHD score was associated with approximately 8% lower GHG emissions (*β*_10pts_ −7.5% (95% CI: −7.8%, −7.1%)). This association was not apparent when red and processed meat intake was excluded from the derivation of the PHD score (S4 Fig).

## Discussion

In this large prospective cohort study, greater adherence to the PHD, which emphasises high consumption of healthful plant foods, and lower consumption of animal foods, was associated with lower incidence of T2D and lower diet-related GHG emissions. The inverse association with incident T2D was robust across population subgroups and in sensitivity analyses. Approximately 12% of incident T2D cases could have been prevented had all participants achieved a PHD score equal to the 80th percentile in the current cohort, equivalent to 85.7 points. In line with our primary findings, the PHD score was inversely associated with markers of adiposity and of glucose and lipid metabolism. In the subset analysis of those with change data available, there was evidence of a higher risk of T2D among those with a large decrease (>10%) in adherence to the PHD over time, compared to those who maintained a stable adherence. Overall, our findings suggest that adherence to the PHD was associated with lower incidence of T2D.

There have been growing calls for a shift towards more sustainable diets to reduce the burden of diet-related noncommunicable diseases, but also to reduce the detrimental environmental impact of unhealthy diets [2,21,22]. The PHD was developed in 2019 to achieve optimal health and reduce diet-related environmental impact. Since then, a few studies have assessed the association between the PHD and T2D risk and presented inconclusive results [4–8]. Two studies that evaluated participants from Scandinavia both reported an approximately 5%–6% lower incidence of T2D per SD higher adherence to the PHD [4,5] unlike our observation of 14% lower incidence of T2D. The difference in this magnitude may have reflected differences in cooking methods for diverse food items. For example, the Scandinavian population consumed fish with minimal cooking with fresh vegetables and whole grains (Nordic diet) [23], while the British population might consume more fried fish with fried potatoes [24]: the latter may strengthen the association of the PHD adherence with T2D incidence. The characteristics associated with high or low adherence to PHD may therefore be diverse, and this was also inferable from the EPIC-Oxford study. The UK’s EPIC-Oxford study showed a greater inverse association than our study, recruiting vegans and vegetarians selectively and other omnivores, creating a greater contrast between high and low PHD adherence groups [8]. By contrast, analyses in both the Mexican Teacher’s Cohort [7] and the UK Biobank [6] yielded weaker or null associations of the PHD with T2D incidence. These inconsistencies may be due to additional components of a plant-based diet in Mexican cuisine and the issue of measurement errors in single-day dietary assessment method in the UK BioBank.

The greater magnitude of an inverse association between the PHD and T2D incidence we observed than in previously published reports [4–7,9] could also be attributed to study design-related factors. There was considerable variation in the methods used to assess adherence to the PHD in previous studies. The majority of those applied a dichotomous scoring to each component of the PHD [4,6–8]. Such categorisation may have limited the statistical power to capture the variability in the levels of adherence to the PHD [25]. Indeed, when we used the dichotomous approach to assess adherence to the PHD, we found that the inverse association between the PHD and T2D incidence became weaker. Additionally, previous studies assessed dietary intake solely at baseline, and thus, were unable to account for within-person dietary variation and other behavioural variations over time [10]. We found that a large decrease (>10%) in adherence to the PHD was associated with higher T2D incidence, but the overall evidence from the analysis of changes was not strong. This could be due to a lesser precision reflecting a smaller sample size, and shorter duration of follow-up. Future studies involving larger cohorts with repeated dietary assessments or a dietary intervention should investigate this further.

Greater adherence to the PHD is characterised by higher consumption of mostly healthful plant foods such as wholegrains, fruits, and vegetables, and lower consumption of animal-derived foods and of less healthful plant foods such as refined grains and sugar-sweetened beverages (SSBs). A meta-analysis reported an inverse association between most of these healthful plant foods and incident T2D, and opposite associations for the less healthful plant foods and animal foods [26]. Thus, the inverse association between the PHD and the risk of T2D may be explained by the beneficial health effects of the healthy food components acting independently, synergistically, or both. The physiological mechanisms proposed to mediate the health benefits of the components of the PHD include improved regulation of bodyweight, insulin and glucose homeostasis, and lipid metabolism [27]. This is consistent with the considerable attenuation of the inverse association between the PHD and T2D risk upon adjustment for cardiometabolic risk markers, as well as the inverse association between the PHD and these markers in our study. Furthermore, our finding that plasma concentrations of antioxidants such as vitamin C and carotenoids explained approximately 32% of the association between the PHD and T2D incidence may have reflected physiological effects such as enhanced antioxidant capacity from these factors [28,29].

With respect to environmental impact, our analyses revealed that greater adherence to the PHD was associated with lower GHG emissions, consistent with previous findings in other countries [30,31]. Notably, in an analysis in which we excluded red and processed meat intake from the derivation of the PHD score, the inverse association between the PHD score and GHG emissions disappeared. Our findings suggest that a reduction in GHG emissions may be particularly dependent on meeting the PHD recommendations of reducing intake of red and processed meat.

Our findings suggest that had all participants in the cohort obtained the 80th percentile PHD score (85.7 points), an estimated 12.3% (95% CI: 9.2%, 15.3%) of incident T2D cases could have been prevented. This is consistent with findings reported previously showing that 70.3% (95% uncertainty interval: 68.8%, 71.8%) of new T2D cases globally were attributed to suboptimal dietary intake, where suboptimal intake of most unhealthy foods was defined as consumption above 0 grams/day [32]. In our cohort, those with a PHD score of at least 85.7 had more varied distribution of intake of some unhealthy foods, and thus, our modelled PAF estimated may be more feasible in the general population. Also, a recent study that used a PHD score with a range of 0–42 reported that, had all participants scored at least 23 points, 12.9% (95% CI: 1.8%, 22.1%) of incident T2D cases could have been averted [5]. These findings suggest that increasing adherence to the PHD, even modestly, may be associated with a considerable reduction in the risk of developing T2D. However, strategies to promote population-wide adoption of the PHD are needed.

A healthy volunteer bias [33] might occur in our study cohort, and thus, we speculate that the number of preventable T2D cases could be higher in the general UK population, because the absolute T2D incidence and variation in the PHD adherence, if measured, would be expected to be higher. Considering that the PHD was purposely developed to reduce the risk of noncommunicable diseases and diet-related environmental impact [3], higher adherence to the PHD may additionally be associated with benefits beyond T2D risk reduction. In our cohort, the magnitude of the inverse association between the PHD and T2D was marginally greater per SD higher score, compared to the Mediterranean diet [34], while both the PHD and the Mediterranean diet showed a largely similar inverse association with diet-related GHG emissions.

We analysed data from a large prospective cohort study, with a long duration of follow-up that measured many potential confounders and dietary intake with repeated measures, which partly accounted for possible dietary and behavioural changes over time. Additionally, scoring of the PHD adherence on a continuous scale enabled us to characterise a greater distinction between participants and validly capture between-individual variability. We ascertained incident T2D with multiple sources of objective information, including physician diagnoses, medication use, retinal screening and biochemical assessment which further minimised potential bias that may arise due to misclassification of T2D diagnosis.

Our study has limitations. Despite the temporality of associations, causal inference is limited by the observational design of our study and the possibility of residual confounding. Self-reported dietary intake and some covariates may have been measured with errors. Possible measurement errors in covariates, such as BMI, may lead to residual confounding in the association between the PHD and T2D incidence, potentially leading to bias in the estimated PAF. We used a single scoring method, but different scoring methods have been used to derive the PHD with different pros and cons [31]. Measurement errors in the subjective assessment of dietary intake may lead to misclassification of adherence to the PHD. For most dietary variables, however, objective assessment is currently unavailable and our use of repeated FFQs together with adjustment for total energy intake and BMI helped to reduce measurement errors and possible misclassification of adherence to the PHD in this study. Our study cohort was predominantly of European-origin white ethnicity, which may limit the generalisability of our findings to non-white populations. Future studies should evaluate these associations in diverse global populations, particularly in East Asian, South Asian, and African countries, where the prevalence of T2D has been increasing. Evidence on the influence of dietary risk factors on T2D incidence, accounting for other context-specific risk factors, remains limited in these regions. We assessed diet-related GHG emissions, but not other indicators of diet-related environmental impact, such as land and water use, and these should be assessed in future research.

Our findings suggest that greater adherence to the PHD is strongly associated with a lower incidence of T2D, and lower GHG emissions. Future research should further confirm these associations in different populations and further evaluate the association of the PHD with multiple health outcomes and environmental impact indicators such as GHG emissions, land use, and freshwater use. In the meantime, the findings of the current research provide support for promotion of the PHD for planetary health and the population-level prevention of T2D.

## Supporting information

S1 Checklist STROBE Statement – Checklist of items that should be included in reports of cohort studies. (DOCX)

S1 Text Assessment of cardiometabolic risk factors. (DOCX)

S2 Text Measurement of plasma vitamin C, carotenoids, and tocopherols. (DOCX)

S3 Text Sensitivity analyses. (DOCX)

S4 Text Secondary analyses. (DOCX)

S1 Table Criteria for assessing adherence to the planetary health diet. TEI,  total energy intake. *Intake of unsaturated fatty acids was used to assess the consumption of added unsaturated oils. ^†^The sum of saturated fatty acids and *trans-*fatty acids intake was used to assess the consumption of added saturated and *trans-*fat.
(DOCX)

S2 Table Association between the planetary health diet score and risk of type 2 diabetes across subgroups of age, sex, BMI, and family history of diabetes: EPIC-Norfolk study, the United Kingdom. Quintile 1 (Q1) represents the lowest adherence to the PHD, while Quintile 5 (Q5) represents the highest adherence. The total possible PHD score ranges from 0 (lowest) to 140 (highest). Data are HR (95% CI). In the continuous analyses, the HRs and 95% CIs were estimated per 10-point higher score of the PHD score. ^1^Model 1: adjusted for age (years) and sex (male or female). ^2^Model 2: adjusted for factors in model 1 plus physical activity (inactive, moderately inactive, moderately active, or active), smoking status (never, former, or current), level of education (primary/none, O-level, A-level, or degree), use of vitamin supplements (yes/no), family history of diabetes (yes/no), alcohol intake (g/d) and energy intake (continuous, kcal/d). ^3^Model 3: adjusted for factors in Model 2 plus body mass index (kg/m^2^). ^4^Model 4: adjusted for factors in Model 3 plus prevalent CVD or cancer. P-interaction was obtained by including comparing a model without and a model with cross-product terms of the PHD score (continuous) and each of the covariates using a likelihood ratio test. BMI, body mass index; CVD, cardiovascular disease; HR, hazard ratio; PHD, planetary health diet; SES, socioeconomic status; T2D, type 2 diabetes.
(DOCX)

S3 Table Association between the planetary health diet and risk of type 2 diabetes in sensitivity analyses. Quintile 1 (Q1) represents the lowest adherence to the PHD, while Quintile 5 (Q5) represents the highest adherence. The total possible PHD score ranges from 0 (lowest) to 140 (highest). Data are HR (95% CI). In the continuous analyses, the HRs and 95% CIs were estimated per 10-point higher score of the PHD score. ^1^Model 1: adjusted for age (years) and sex (male or female). ^2^Model 2: adjusted for factors in model 1 plus physical activity (inactive, moderately inactive, moderately active, or active), smoking status (never, former, or current), level of education (primary/none, O-level, A-level, or degree), use of vitamin supplements (yes/no), family history of diabetes (yes/no), alcohol intake (g/d) and energy intake (continuous, kcal/d). ^3^Model 3: adjusted for factors in Model 2 plus body mass index (kg/m^2^). ^4^Model 4: adjusted for factors in Model 3 plus prevalent CVD or cancer. ^5^Model 5: adjusted for factors in Model 4 plus use of hormone replacement therapy at baseline (females only). ^6^Model 5: adjusted for factors in Model 4 plus the Mediterranean diet score. ^7^Model 5: adjusted for factors in Model 4 plus cardiometabolic risk markers, i.e., waist circumference, systolic and diastolic blood pressure, HbA1c, plasma triglycerides, total cholesterol, low-density lipoprotein cholesterol, high-density lipoprotein cholesterol, and C-reactive protein. *HR (95% CI) per unit higher score. BMI, body mass index; CRM, cardiometabolic risk markers; CVD, cardiovascular disease; FFQ, food frequency questionnaire; HbA1c, glycated haemoglobin; HR, hazard ratio; PHD, planetary health diet; SES, socioeconomic status; T2D, type 2 diabetes.
(DOCX)

S4 Table Evaluation of the potential effect of nutritional biomarkers on the association between the planetary health diet and risk of type 2 diabetes. ^1^For each nutritional biomarker, the analyses were performed for a subsample of participants who had data for the biomarker, hence the sample sizes vary for each biomarker. All Cox models were adjusted for age (years), sex (male or female), physical activity (inactive, moderately inactive, moderately active, active), energy intake (kcal/d), smoking status (never, former, or current), level of education (primary/none, O-level, A-level, or degree), use of vitamin supplements (yes/no), family history of diabetes (yes/no), alcohol intake (g/d), and BMI (kg/m^2^), and prevalent CVD or cancer. ^2^HR (95% CI) of the association between the PHD and risk of T2D; HRs (95% CIs) estimate the risk of type 2 diabetes per 10-points higher PHD score. ^3^HR (95% CI) of the association between the nutritional biomarker and risk of T2D, in a model that additionally adjusted for the PHD; the HR (95% CI) estimate the risk of type 2 diabetes per-SD of nutritional biomarker. ^4^HR (95%) CI of the association between the PHD and risk of T2D, in a model adjusted for the nutritional biomarker. Proportion of the PHD-T2D association explained by the nutritional biomarker. BMI, body mass index; PHD, planetary health diet; HR, hazard ratio; SD, standard deviation; T2D, type 2 diabetes.
(DOCX)

S1 Fig Flowchart of EPIC-Norfolk participants included in the analyses. FFQ, food frequency questionnaire.
(TIF)

S2 Fig Association between the planetary health diet and type 2 diabetes risk after iteratively omitting one component from the calculation of the diet score. Data are HRs and 95% CIs per 10-point higher PHD score obtained by fitting the most adjusted Cox model. The model adjusted for age (years), sex (male or female), plus physical activity (inactive, moderately inactive, moderately active, or active), smoking status (never, former, or current), level of education (primary/none, O-level, A-level, or degree), use of vitamin supplements (yes/no), family history of diabetes (yes/no), alcohol intake (g/d), energy intake (continuous, kcal/d), body mass index (kg/m2), prevalent CVD or cancer, and the PHD component excluded from the calculation of the PHD score. BMI, body mass index; CVD, cardiovascular disease; HR, hazard ratio; PHD, planetary health diet.
(TIF)

S3 Fig Multivariable-adjusted association of the planetary health diet (per 10 points) with cardiometabolic risk markers. The cardiometabolic risk factors were log-transformed prior to the analysis, and the beta estimates were back-transformed before being plotted. Thus, a null effect corresponds to an estimate of 1.00 (dashed vertical line). The models were adjusted for age (years), sex(male or female), physical activity (inactive, moderately inactive, moderately active, or active), smoking status (never, former, or current), level of education (primary/none, O-level, A-level, or degree), use of multivitamin supplements (yes/no), family history of diabetes (yes/no), alcohol intake (g/d), energy intake (kcal/d), and BMI (kg/m^2^) (except when BMI was the outcome). ALT, Alanine aminotransferase; AST, Aspartate aminotransferase; BMI, body mass index; CRP, C-reactive protein; GGT, Gamma-glutamyltransferase; HbA1c, glycated haemoglobin; HDL, high density lipoprotein; LDL, low density lipoprotein.
(TIF)

S4 Fig Multivariable-adjusted association of the planetary health diet score with diet-related greenhouse gas emissions after iteratively excluding one component of the score. The PHD score was modelled as quintiles, and the models were adjusted for age (years), sex (male or female), alcohol intake (g/d), energy intake (kcal/d), and the score for the PHD food component excluded from the derivation of the score. The models included a random intercept for participant identifier to account to the repeated measurements of the PHD score and greenhouse gas (GHG) emissions. The estimates represent the expected percentage differences (95% CI) in the GHG emissions (kgCO_2_eq/d) for participants in quintile 5 compared to those in quintile 1 (reference category). The red vertical line is the reference line for no association between the PHD score and GHG emissions. The blue vertical line is positioned at the value of the point estimate obtained from the linear regression analysis for the association between the PHD score (based on all components) and GHG emissions, adjusting for age, sex, alcohol intake, and energy intake. GHG, greenhouse gas; PHD, planetary health diet; Q1and Q5, quintiles 1 and 5, respectively.
(TIF)

## Abbreviations

**:**
ALT: alanine aminotransferase
AST: aspartate aminotransferase
CIs: confidence intervals
CRP: C-reactive protein
CVD: cardiovascular disease
DBP: diastolic blood pressure
FFQ: food frequency questionnaire
GGT: gamma-glutamyl transferase
GHG: greenhouse gas
HDL-cholesterol: high-density lipoprotein cholesterol
HRs: hazard ratios
LDL-cholesterol: low-density lipoprotein cholesterol
PAF: population attributable fraction
PHD: planetary health diet
SBP: systolic blood pressure
T2D: type 2 diabetes
WC: waist circumference
