# Supplementary material for: The association of the planetary health diet with type 2 diabetes incidence and greenhouse gas emissions: Findings from the EPIC-Norfolk prospective cohort study
Source: PLoS Med. 2025 Sep 16;22(9):e1004633. doi: 10.1371/journal.pmed.1004633 (PMC12440362; doi:10.1371/journal.pmed.1004633)
Supplement: S1 Checklist — (DOCX) [file pmed.1004633.s001.docx]

STROBE Statement—Checklist of items that should be included in reports of ***cohort studies***

|  | Item No | Recommendation | Page No |
| --- | --- | --- | --- |
| **Title and abstract** | 1 | (*a*) Indicate the study’s design with a commonly used term in the title or the abstract | 1, 3, title page |
|  |  | (*b*) Provide in the abstract an informative and balanced summary of what was done and what was found | 3, abstract section |
| Introduction | | | |
| Background/rationale | 2 | Explain the scientific background and rationale for the investigation being reported | 6, paragraphs 2-3 |
| Objectives | 3 | State specific objectives, including any prespecified hypotheses | 7, last paragraph of introduction |
| Methods | | | |
| Study design | 4 | Present key elements of study design early in the paper | 7, study design and participants section |
| Setting | 5 | Describe the setting, locations, and relevant dates, including periods of recruitment, exposure, follow-up, and data collection | 7, study design and participants section |
| Participants | 6 | (*a*) Give the eligibility criteria, and the sources and methods of selection of participants. Describe methods of follow-up | 7, study design and participants section |
|  |  | (*b*) For matched studies, give matching criteria and number of exposed and unexposed | N/A |
| Variables | 7 | Clearly define all outcomes, exposures, predictors, potential confounders, and effect modifiers. Give diagnostic criteria, if applicable | 8-10, subsections 2-5 of the methods section |
| Data sources/ measurement | 8* | For each variable of interest, give sources of data and details of methods of assessment (measurement). Describe comparability of assessment methods if there is more than one group | 8-10, subsections 2-5 of the methods section |
| Bias | 9 | Describe any efforts to address potential sources of bias | 9, statistical analysis section |
| Study size | 10 | Explain how the study size was arrived at | N/A |
| Quantitative variables | 11 | Explain how quantitative variables were handled in the analyses. If applicable, describe which groupings were chosen and why | 10, statistical analysis section |
| Statistical methods | 12 | (*a*) Describe all statistical methods, including those used to control for confounding | 10-12, statistical analysis section |
|  |  | (*b*) Describe any methods used to examine subgroups and interactions | 11-12, Effect modification, subgroup and sensitivity analyses section |
|  |  | (*c*) Explain how missing data were addressed | N/A |
|  |  | (*d*) If applicable, explain how loss to follow-up was addressed | N/A |
|  |  | (*e*) Describe any sensitivity analyses | 11-12, Effect modification, subgroup and sensitivity analyses section, and Supplementary Text 3 |
| Results | | |  |
| Participants | 13* | (a) Report numbers of individuals at each stage of study—eg numbers potentially eligible, examined for eligibility, confirmed eligible, included in the study, completing follow-up, and analysed | 7, study design and participants subsection |
|  |  | (b) Give reasons for non-participation at each stage | 7, study design and participants subsection |
|  |  | (c) Consider use of a flow diagram | 7, study design and participants subsection |
| Descriptive data | 14* | (a) Give characteristics of study participants (eg demographic, clinical, social) and information on exposures and potential confounders | 13, characteristics of participants section |
|  |  | (b) Indicate number of participants with missing data for each variable of interest | 7, study design and participants subsection |
|  |  | (c) Summarise follow-up time (eg, average and total amount) | 13, characteristics of participants section |
| Outcome data | 15* | Report numbers of outcome events or summary measures over time | 18, Results section Table 2 |

| Main results | 16 | (*a*) Give unadjusted estimates and, if applicable, confounder-adjusted estimates and their precision (eg, 95% confidence interval). Make clear which confounders were adjusted for and why they were included | 18, Results section Table 2 |
| --- | --- | --- | --- |
|  |  | (*b*) Report category boundaries when continuous variables were categorized | 18, Results section Table 2 |
|  |  | (*c*) If relevant, consider translating estimates of relative risk into absolute risk for a meaningful time period | 18, Results section Table 2 |
| Other analyses | 17 | Report other analyses done—eg analyses of subgroups and interactions, and sensitivity analyses | 11-12, described with reference to supporting information |
| Discussion | | | |
| Key results | 18 | Summarise key results with reference to study objectives | 23, first paragraph |
| Limitations | 19 | Discuss limitations of the study, taking into account sources of potential bias or imprecision. Discuss both direction and magnitude of any potential bias | 26-27, strengths and limitations subsection |
| Interpretation | 20 | Give a cautious overall interpretation of results considering objectives, limitations, multiplicity of analyses, results from similar studies, and other relevant evidence | 23-26, discussion section |
| Generalisability | 21 | Discuss the generalisability (external validity) of the study results | 23-27, discussion section |
| Other information | | | |
| Funding | 22 | Give the source of funding and the role of the funders for the present study and, if applicable, for the original study on which the present article is based | Funding section |

*Give information separately for exposed and unexposed groups.

**Note:** An Explanation and Elaboration article discusses each checklist item and gives methodological background and published examples of transparent reporting. The STROBE checklist is best used in conjunction with this article (freely available on the Web sites of PLoS Medicine at http://www.plosmedicine.org/, Annals of Internal Medicine at http://www.annals.org/, and Epidemiology at http://www.epidem.com/). Information on the STROBE Initiative is available at http://www.strobe-statement.org.
