# Supplementary material for: The association of the planetary health diet with type 2 diabetes incidence and greenhouse gas emissions: Findings from the EPIC-Norfolk prospective cohort study
Source: PLoS Med. 2025 Sep 16;22(9):e1004633. doi: 10.1371/journal.pmed.1004633 (PMC12440362; doi:10.1371/journal.pmed.1004633)
Supplement: S1 Text — (DOCX) [file pmed.1004633.s002.docx]

**S1 Text. Assessment of cardiometabolic risk factors**

Body weight, height, and waist circumference (WC) were measured with participants wearing light clothes and no shoes or socks. Height was measured to the nearest 0.1 cm with a stadiometer after inhalation, with the participant standing as tall and straight as possible with feet together. Weight was also recorded to the nearest 0.1 kg. Body mass index (BMI, kg/m^2^) was calculated as the weight in kilograms (kg) divided by the square of the height in meters (m^2^). WC was measured with a D loop, non-stretch fiberglass tape after the end of a normal expiration and at the narrowest point, i.e., the circumference between the lower rib margin and the iliac crest. If the minimum circumference was not identifiable, the WC was measured at the level of the nave.

Blood pressure was measured twice in the right arm with an Accutorr sphygmomanometer (Datascope, Huntingdon, UK) after the participant had been seated for a three-minute rest. The mean the two measurements was used for the analysis [1].

The concentrations of blood biomarkers were measured on non-fasting blood samples obtained by venipuncture. Blood samples were assayed at the department of clinical biochemistry at the University of Cambridge, UK. Serum concentrations of total cholesterol, high-density lipoprotein cholesterol and triglycerides were measured with the RA 1000 Technicon analyser (Bayer Diagnostics, Basingstoke). The concentration of low-density lipoprotein cholesterol was calculated using the Friedewald formula, except when the concentration of triglyceride exceeded 4 mmol/l [1]. Serum C-reactive protein (CRP) concentration was measured with a high sensitivity CRP test. Glycated haemoglobin (HbA1c, %) was measured on fresh EDTA samples by using high-performance liquid chromatography (HPLC) (BioRad Diamat Automated Glycosylated Haemoglobin Analyser, Hemel Hempstead, United Kingdom).

Glucose, Gamma-glutamyltransferase (GGT), Alanine aminotransferase (ALT), and Aspartate aminotransferase (AST) were assayed on the Olympus AU640 Chemistry Analyser (Beckman Coulter, USA).

**References**

1. Silvia MOT, Sheila B, Ailsa W, Robert L, Suzy O, Nicholas D, et al. Frequency of eating and concentrations of serum cholesterol in the Norfolk population of the European prospective investigation into cancer (EPIC-Norfolk): cross sectional study. BMJ. 2001;323(7324):1286.
