# Supplementary material for: The association of the planetary health diet with type 2 diabetes incidence and greenhouse gas emissions: Findings from the EPIC-Norfolk prospective cohort study
Source: PLoS Med. 2025 Sep 16;22(9):e1004633. doi: 10.1371/journal.pmed.1004633 (PMC12440362; doi:10.1371/journal.pmed.1004633)
Supplement: S2 Text — (DOCX) [file pmed.1004633.s003.docx]

# S2 Text. Measurement of plasma vitamin C, carotenoids and tocopherols

Plasma vitamin C was measured in plasma prepared from blood collected into citrated tubes at baseline as described previously. After collection, the blood samples were stored overnight in a black box at 4–7 °C, centrifuged at 2100 g for 15 min at 4 °C, stabilized in a standardized volume of metaphosphoric acid, followed by storage at -70°C. A fluorometric assay was used to estimate plasma vitamin C concentrations (μmol/l) within one week of sampling. The range of the coefficient of variation was 2.7–6.2 [1].

Plasma concentrations of the six carotenoids i.e., lutein, zeaxanthin, beta-cryptoxanthin, lycopene, alpha-carotene and beta-carotene, as well as alpha-tocopherol and gamma-tocopherol were measured at the International Agency for Research on Cancer (IARC) using a reverse-phase an HPLC method on an HPLC-1100 system (Hewlett Packard, Wilmington, IL, USA) with a C18-Adsorbosphere column (Alltech, Deerfield, IL, USA). As discussed in detail previously [2, 3], 200μl plasma samples were thawed and deproteinated with alcohol, extracted with hexane, dried under vacuum, and reconstituted with 300 μl of a mixture of methanol (88%)/ ethanol (10%)/ hexane (2%). Internal samples were run with each sample, and in each batch an external calibration was performed using the standard solutions at eight different concentrations. The coefficients of variation were: 10.1% for α-carotene, 5.5% for β-carotene, 6.5% for β-cryptoxanthin, 4.3% for lutein, 7.4% for lycopene, 8.4% for zeaxanthin, 5.6% for alpha-tocopherol and 8.4% for gamma-tocopherol.

# References

1. Cooper AJ, Sharp SJ, Luben RN, Khaw KT, Wareham NJ, Forouhi NG. The association between a biomarker score for fruit and vegetable intake and incident type 2 diabetes: the EPIC-Norfolk study. Eur J Clin Nutr. 2015;69(4):449-54. Epub 2014/11/13. PubMed PMID: 25387899; PubMed Central PMCID: PMCPMC4704139.

2. Jenab M, Riboli E, Ferrari P, Friesen M, Sabate J, Norat T, et al. Plasma and dietary carotenoid, retinol and tocopherol levels and the risk of gastric adenocarcinomas in the European prospective investigation into cancer and nutrition. Br J Cancer. 2006;95(3):406-15.

3. Steghens JP, van Kappel AL, Riboli E, Collombel C. Simultaneous measurement of seven carotenoids, retinol and alpha-tocopherol in serum by high-performance liquid chromatography. J Chromatogr B Biomed Sci Appl. 1997;694(1):71-81. Epub 1997/06/20. PubMed PMID: 9234850.
