# Supplementary material for: The association of the planetary health diet with type 2 diabetes incidence and greenhouse gas emissions: Findings from the EPIC-Norfolk prospective cohort study
Source: PLoS Med. 2025 Sep 16;22(9):e1004633. doi: 10.1371/journal.pmed.1004633 (PMC12440362; doi:10.1371/journal.pmed.1004633)
Supplement: S3 Text — (DOCX) [file pmed.1004633.s004.docx]

# S3 Text. Sensitivity analyses

To minimise reverse causation, we excluded participants who had HbA1c >6.5% at baseline or those who developed T2D within the first two years of follow-up. Furthermore, to explore the influence of comorbidities at baseline, we excluded those with prevalent CVD or cancer. To estimate the effect of the PHD independent of established healthy dietary patterns, we additionally adjusted for the Mediterranean diet score [1]. We further evaluated the influence of each component of the PHD on its overall effect estimate by iteratively re-calculating the PHD score after excluding one component and evaluating the association between the re-calculated PHD score and T2D risk, adjusting for the excluded component. We additionally assessed the influence of potential energy misreporting by repeated the main analysis after excluding participants who were likely to report invalid energy intakes, defined as <600 kcal/d or >3,500 kcal/d for women, and <800 kcal/d or >4,200 kcal/d for men [2]. To evaluate the influence of missing data on our primary estimates, missing covariate data were imputed by multiple imputation using chained equations in 10 datasets, and estimates were pooled across the 10 datasets using Rubin’s rule [3].

Furthermore, we adjusted for several cardiometabolic risk markers, i.e., WC, systolic and diastolic blood pressure, HbA1c, plasma triglycerides, total cholesterol, low-density lipoprotein cholesterol (LDL-cholesterol), high-density lipoprotein cholesterol (HDL-cholesterol), and C-reactive protein (CRP) to estimate the association of the PHD with T2D independent of these risk factors. Additionally, in an analysis restricted to women only, we adjusted for the use of hormone replacement therapy at baseline (HRT). We further assessed the association between the PHD and the risk of T2D using the baseline data only for the PHD and covariates to examine the long-term association at the expense of not accounting for within-person variability of time-varying factors.

# References

1. Sofi F, Macchi C, Abbate R, Gensini GF, Casini A. Mediterranean diet and health status: an updated meta-analysis and a proposal for a literature-based adherence score. Public Health Nutr. 2014;17(12):2769-82. Epub 2014/01/31. PubMed PMID: 24476641.

2. Willett W. Nutritional epidemiology: Oxford university press; 2012.

3. White IR, Royston P, Wood AM. Multiple imputation using chained equations: Issues and guidance for practice. Stat Med. 2011;30(4):377-99. Epub 2011/01/13. PubMed PMID: 21225900.
