# Supplementary material for: The association of the planetary health diet with type 2 diabetes incidence and greenhouse gas emissions: Findings from the EPIC-Norfolk prospective cohort study
Source: PLoS Med. 2025 Sep 16;22(9):e1004633. doi: 10.1371/journal.pmed.1004633 (PMC12440362; doi:10.1371/journal.pmed.1004633)
Supplement: S4 Text — (DOCX) [file pmed.1004633.s005.docx]

**S4 Text. Secondary analyses**

Most previous studies calculated the PHD assigning 0 or 1 point to each of the PHD component dichotomously [1-4]. We took the same dichotomous approach to evaluate the influence, by awarding a score of one for adherence to each PHD component (0.5 for soy and non-soy legumes), and a score of zero for non-adherence. The scores were then summed across the 15 components to yield a score that ranged from zero (lowest) to 14 (highest).

Many plant foods are abundant in vitamin C, vitamin E and carotenoids [5], and plasma concentrations of these biomarkers have been used as objective biomarkers of fruit and vegetable intake [6, 7]. Thus, we investigated whether the association between the PHD score and T2D risk was explained by pre-selected available nutritional biomarkers, including plasma vitamin C, six carotenoids (alpha- and beta-carotene, lycopene, lutein, zeaxanthin, and cryptoxanthin), and alpha- and gamma-tocopherol. The nine nutritional biomarkers were preselected based on their reported positive association with the intake of plant foods such as fruits, vegetables and legumes [8], as well as the availability of these biomarkers in the EPIC-Norfolk subsample, with varied sample sizes for each biomarker (n=6793–20 887). All biomarkers were winsorised (top and bottom 10%) and standardised before the analyses [9]. We repeated the main analysis with and without adjustment for those biomarkers and calculated the proportion (%) of the PHD-T2D association attenuated (or strengthened) with the adjustment for those biomarkers as (HR_1_ - HR_2_) / (HR_1_ - 1) [10], where HR_1_ was the estimate without the adjustment, and HR_2_, with the adjustment. The 95% CI of the estimate was calculated using a bootstrap with 1000 iterations. We repeated the same procedure for each of the available biomarkers. This analysis was restricted to data collected at baseline, as data for the nutritional biomarkers were mostly available only at baseline. We further examined associations between the PHD score and cardiometabolic risk markers, using multivariable linear regression models. As a dependent variable, we evaluated each of the clinical risk factors described above. The models were adjusted for the covariates used in the main Cox regression analysis, except for BMI when BMI was the outcome. The cardiometabolic markers were measured at baseline, thus these analyses were cross-sectional at baseline.

**References**

1. Knuppel A, Papier K, Key TJ, Travis RC. EAT-Lancet score and major health outcomes: the EPIC-Oxford study. Lancet. 2019;394(10194):213-4. Epub 2019/06/27. PubMed PMID: 31235280.

2. Langmann F, Ibsen DB, Tjønneland A, Olsen A, Overvad K, Dahm CC. Adherence to the EAT-Lancet diet is associated with a lower risk of type 2 diabetes: the Danish Diet, Cancer and Health cohort. Eur J Nutr. 2023;62(3):1493-502. Epub 2023/01/24. PubMed PMID: 36688993.

3. López GE, Batis C, González C, Chávez M, Cortés-Valencia A, López-Ridaura R, et al. EAT-Lancet Healthy Reference Diet score and diabetes incidence in a cohort of Mexican women. Eur J Clin Nutr. 2023;77(3):348-55. Epub 2022/12/06. PubMed PMID: 36471166.

4. Xu C, Cao Z, Yang H, Hou Y, Wang X, Wang Y. Association Between the EAT-Lancet Diet Pattern and Risk of Type 2 Diabetes: A Prospective Cohort Study. Front Nutr. 2021;8:784018. Epub 2022/02/01. PubMed PMID: 35096931; PubMed Central PMCID: PMCPMC8795697.

5. Carlsen MH, Halvorsen BL, Holte K, Bøhn SK, Dragland S, Sampson L, et al. The total antioxidant content of more than 3100 foods, beverages, spices, herbs and supplements used worldwide. Nutrition Journal. 2010;9(1):3.

6. Cooper AJ, Sharp SJ, Luben RN, Khaw KT, Wareham NJ, Forouhi NG. The association between a biomarker score for fruit and vegetable intake and incident type 2 diabetes: the EPIC-Norfolk study. Eur J Clin Nutr. 2015;69(4):449-54. Epub 2014/11/13. PubMed PMID: 25387899; PubMed Central PMCID: PMCPMC4704139.

7. Zheng JS, Sharp SJ, Imamura F, Chowdhury R, Gundersen TE, Steur M, et al. Association of plasma biomarkers of fruit and vegetable intake with incident type 2 diabetes: EPIC-InterAct case-cohort study in eight European countries. Bmj. 2020;370:m2194. Epub 2020/07/10. PubMed PMID: 32641421.

8. Al-Delaimy WK, Ferrari P, Slimani N, Pala V, Johansson I, Nilsson S, et al. Plasma carotenoids as biomarkers of intake of fruits and vegetables: individual-level correlations in the European Prospective Investigation into Cancer and Nutrition (EPIC). Eur J Clin Nutr. 2005;59(12):1387-96. Epub 2005/09/15. PubMed PMID: 16160702.

9. Baron RM, Kenny DA. The moderator-mediator variable distinction in social psychological research: conceptual, strategic, and statistical considerations. J Pers Soc Psychol. 1986;51(6):1173-82. Epub 1986/12/01. PubMed PMID: 3806354.

10. Murray CJL, Aravkin AY, Zheng P, Abbafati C, Abbas KM, Abbasi-Kangevari M, et al. Global burden of 87 risk factors in 204 countries and territories, 1990-2019: a systematic analysis for the Global Burden of Disease Study 2019. The Lancet. 2020;396(10258):1223-49.
