# Supplementary material for: The association of the planetary health diet with type 2 diabetes incidence and greenhouse gas emissions: Findings from the EPIC-Norfolk prospective cohort study
Source: PLoS Med. 2025 Sep 16;22(9):e1004633. doi: 10.1371/journal.pmed.1004633 (PMC12440362; doi:10.1371/journal.pmed.1004633)
Supplement: S1 Table — TEI, total energy intake. *Intake of unsaturated fatty acids was used to assess the consumption of added unsaturated oils. †The sum of saturated fatty acids and trans-fatty acids intake was used to assess the consumption of added saturated and trans-fat. (DOCX) [file pmed.1004633.s006.docx]

| **S1 Table. Criteria for assessing adherence to the Planetary Health Diet** | | | | | | |
| --- | --- | --- | --- | --- | --- | --- |
| **PHD**  **Component** | **Food items**  **(EPIC-Norfolk study)** | **EAT-Lancet reference diet  (for 2,500 kcal/d)** | | **Criteria for scoring for PHD (range 0-140)**  **based on amount consumed (g/d)** | | |
|  |  | **g/day (uncertainty range)** | **kcal/day** | **Min score (0)** | **Max score (10)** | **Weight in total score** |
| Whole grains (dry weight) | Wholegrain cereals, porridge, wholemeal bread, wholemeal pasta, brown bread, crisp bread, and brown rice | 232 (0-60% of TEI) | 811 | 0 | Women: ≥ 75  Men: ≥ 90 | 1 |
| Starchy vegetables | Potatoes | 50 (0-100) | 39 | ≥ 200 | ≤ 50 | 1 |
| Vegetables | Carrots, spinach, broccoli, sprouts, cabbage, leeks, peppers, tomatoes, green salad, watercress, vegetable soup, onions, garlic, mushrooms, parsnips, marrow, beetroot, beansprouts, coleslaw, pickles, sweetcorn, and cauliflower | 300 (200-600) | 78 | 0 | ≥ 300 | 1 |
| Fruits | Apples, pears, oranges, grapefruit, bananas, grapes, melons, peaches, strawberries, dried fruit, avocado, and tinned fruits | 200 (100-300) | 126 | 0 | ≥ 200 | 1 |
| Dairy foods | Milk, single cream, double cream, low-fat yoghurt, full-fat yoghurt, dairy dessert, cheese, and cottage cheese | 250 (0-500) | 153 | ≥ 1000 | ≤ 250 | 1 |
| Red/processed meat | Beef, burger, pork, lamb, chicken, bacon, ham, corned beef, sausages, and liver | 14 (0-28) | 30 | ≥ 300 | ≤ 14 | 1 |
| Chicken and other poultry | Chicken | 29 (0-58) | 62 | ≥ 58 | ≤ 29 | 1 |
| Eggs | Eggs | 12 (0-25) | 19 | ≥ 120 | ≤ 12 | 1 |
| Fish | Fried fish, shellfish, fish fingers, white fish, oily fish, and roe | 28 (0-100) | 40 | 0 | ≥ 28 | 1 |
| Nuts | Nuts and peanut butter | 50 (0-75) | 291 | 0 | ≥ 50 | 1 |
| Non-soy legumes | Peas, green beans, beans, and lentils | 75 (0-150) | 284 | 0 | ≥100 | 0.5 |
| Soy foods | Tofu | 25 (0-50) | 112 | 0 | ≥50 | 0.5 |
| Added fat – unsaturated oils^*^ | Sum of *cis-*monounsaturated and *cis-*polyunsaturated FAs | 40 (20-80) | 354 (14.16% of TEI) | ≤ 3.5% of TEI | ≥ 21% of TEI | 1 |
| Added fat – saturated oils, trans-fat^†^ | Sum of saturated FAs and *trans*-FAs | 11.8 (0-11.8) | 96 (3.8% of TEI) | ≥ 10% of TEI | 0% of TEI | 1 |
| Added sugar and fruit juice | Fizzy drinks, fruit juice, squash, sugar | 31 (0-31) | 120 (4.8% of TEI) | ≥25% of TEI | <5% of TEI | 1 |

TEI= total energy intake. *Intake of unsaturated fatty acids was used to assess the consumption of added unsaturated oils. ^†^The sum of saturated fatty acids and trans-fatty acids intake was used to assess the consumption of added saturated and trans-fat.
