# Supplementary material for: The association of the planetary health diet with type 2 diabetes incidence and greenhouse gas emissions: Findings from the EPIC-Norfolk prospective cohort study
Source: PLoS Med. 2025 Sep 16;22(9):e1004633. doi: 10.1371/journal.pmed.1004633 (PMC12440362; doi:10.1371/journal.pmed.1004633)
Supplement: S2 Table — Quintile 1 (Q1) represents the lowest adherence to the PHD, while Quintile 5 (Q5) represents the highest adherence. The total possible PHD score ranges from 0 (lowest) to 140 (highest). Data are HR (95% CI). In the continuous analyses, the HRs and 95% CIs were estimated per 10-point higher score of the PHD score. 1Model 1: adjusted for age (years) and sex (male or female). 2Model 2: adjusted for factors in model 1 plus physical activity (inactive, moderately inactive, moderately active, or active), smoking status (never, former, or current), level of education (primary/none, O-level, A-level, or degree), use of vitamin supplements (yes/no), family history of diabetes (yes/no), alcohol intake (g/d) and energy intake (continuous, kcal/d). 3Model 3: adjusted for factors in Model 2 plus body mass index (kg/m2). 4Model 4: adjusted for factors in Model 3 plus prevalent CVD or cancer. P-interaction was obtained by including comparing a model without and a model with cross-product terms of the PHD score (continuous) and each of the covariates using a likelihood ratio test. BMI, body mass index; CVD, cardiovascular disease; HR, hazard ratio; PHD, planetary health diet; SES, socioeconomic status; T2D, type 2 diabetes. (DOCX) [file pmed.1004633.s007.docx]

| **S2 Table. Association between the Planetary Health Diet Score and risk of type 2 diabetes across subgroups of age, sex, BMI and family history of diabetes: EPIC-Norfolk study, the United Kingdom** | | | | | | | | |
| --- | --- | --- | --- | --- | --- | --- | --- | --- |
|  | Q1 | Q2 | Q3 | Q4 | Q5 | p-trend | HR per 10-points | p-interaction |
| **Age, y** |  |  |  |  |  |  |  |  |
| **<60** |  |  |  |  |  |  |  |  |
| n | 2,661 | 2,556 | 2,499 | 2,418 | 2,579 |  |  |  |
| PHD score, range | 33.9 - 68.4 | 68.4 - 74.7 | 74.7 - 79.8 | 79.8 - 85.7 | 85.7 - 116.8 |  |  |  |
| n cases (T2D)/person-years | 556/55,794 | 361/55,429 | 327/54,914 | 282/53,492 | 303/57,472 |  |  |  |
| adj. for age and sex^1^ | 1 | 0.69 (0.61, 0.79) | 0.64 (0.56, 0.74) | 0.56 (0.48, 0.65) | 0.56 (0.48, 0.64) |  |  |  |
| + SES/ behaviours^2^ | 1 | 0.71 (0.62, 0.81) | 0.68 (0.59, 0.78) | 0.60 (0.52, 0.70) | 0.61 (0.53, 0.71) |  |  |  |
| + BMI^3^ | 1 | 0.72 (0.63, 0.82) | 0.68 (0.59, 0.78) | 0.62 (0.54, 0.72) | 0.68 (0.58, 0.78) |  |  |  |
| + prevalent CVD or cancer^4^ | 1 | 0.72 (0.63, 0.83) | 0.68 (0.59, 0.78) | 0.63 (0.54, 0.73) | 0.67 (0.58, 0.78) | <0.0001 | 0.85 (0.81, 0.89) |  |
| **>60** |  |  |  |  |  |  |  |  |
| n | 2,084 | 2,189 | 2,243 | 2,327 | 2,166 |  |  |  |
| PHD score, range | 39.9 - 68.4 | 68.4 - 74.7 | 74.7 - 79.8 | 79.8 - 85.7 | 85.7 - 117.8 |  |  |  |
| n cases (T2D)/person-years | 362/32,723 | 394/34,916 | 343/37,793 | 311/40,236 | 257/38,316 |  |  |  |
| adj. for age and sex^1^ | 1 | 1.05 (0.91, 1.20) | 0.79 (0.68, 0.91) | 0.72 (0.62, 0.84) | 0.65 (0.55, 0.76) |  |  |  |
| + SES/ behaviours^2^ | 1 | 1.06 (0.93, 1.22) | 0.83 (0.71, 0.96) | 0.76 (0.65, 0.88) | 0.68 (0.58, 0.80) |  |  |  |
| + BMI^3^ | 1 | 1.04 (0.90, 1.20) | 0.82 (0.71, 0.95) | 0.77 (0.66, 0.90) | 0.71 (0.60, 0.84) |  |  |  |
| + prevalent CVD or cancer^4^ | 1 | 1.04 (0.91, 1.20) | 0.82 (0.71, 0.96) | 0.77 (0.66, 0.89) | 0.70 (0.60, 0.83) | <0.0001 | 0.87 (0.83, 0.92) | 0.16 |
| **Sex** |  |  |  |  |  |  |  |  |
| **Male** |  |  |  |  |  |  |  |  |
| n | 2,861 | 2,270 | 2,107 | 1,832 | 1,592 |  |  |  |
| PHD score, range | 33.9 - 68.4 | 68.4 - 74.7 | 74.7 - 79.8 | 79.8 - 85.7 | 85.7 - 116.0 |  |  |  |
| n cases (T2D)/person-years | 595/51,672 | 398/41,247 | 328/38,947 | 262/34,235 | 206/30,657 |  |  |  |
| adj. for age and sex^1^ | 1 | 0.88 (0.77, 0.99) | 0.70 (0.61, 0.80) | 0.64 (0.55, 0.74) | 0.59 (0.51, 0.70) |  |  |  |
| + SES/ behaviours^2^ | 1 | 0.89 (0.79, 1.01) | 0.75 (0.65, 0.86) | 0.68 (0.58, 0.78) | 0.64 (0.55, 0.76) |  |  |  |
| + BMI^3^ | 1 | 0.88 (0.78, 1.00) | 0.72 (0.62, 0.82) | 0.66 (0.57, 0.76) | 0.65 (0.55, 0.77) |  |  |  |
| + prevalent CVD or cancer^4^ | 1 | 0.88 (0.78, 1.00) | 0.72 (0.62, 0.82) | 0.65 (0.56, 0.76) | 0.64 (0.54, 0.75) | <0.0001 | 0.84 (0.80, 0.88) |  |
| **Female** |  |  |  |  |  |  |  |  |
| n | 1,884 | 2,475 | 2,635 | 2,913 | 3,153 |  |  |  |
| PHD score, range | 41.7 - 68.4 | 68.4 - 74.7 | 74.7 - 79.8 | 79.8 - 85.7 | 85.7 - 117.8 |  |  |  |
| n cases (T2D)/person-years | 323/36,846 | 357/49,097 | 342/53,761 | 331/59,493 | 354/65,132 |  |  |  |
| adj. for age and sex^1^ | 1 | 0.80 (0.69, 0.92) | 0.70 (0.61, 0.81) | 0.61 (0.53, 0.72) | 0.59 (0.51, 0.68) |  |  |  |
| + SES/ behaviours^2^ | 1 | 0.82 (0.71, 0.95) | 0.73 (0.63, 0.85) | 0.66 (0.57, 0.77) | 0.64 (0.55, 0.75) |  |  |  |
| + BMI^3^ | 1 | 0.82 (0.71, 0.95) | 0.75 (0.65, 0.87) | 0.70 (0.60, 0.82) | 0.70 (0.60, 0.82) |  |  |  |
| + prevalent CVD or cancer^4^ | 1 | 0.83 (0.71, 0.96) | 0.75 (0.65, 0.88) | 0.70 (0.60, 0.82) | 0.71 (0.61, 0.82) | <0.0001 | 0.89 (0.84, 0.93) | 0.12 |
| **BMI, kg/m^2^** |  |  |  |  |  |  |  |  |
| **<25** |  |  |  |  |  |  |  |  |
| n | 1,739 | 1,739 | 1,761 | 1,892 | 2,180 |  |  |  |
| PHD score, range | 33.9 - 68.4 | 68.4 - 74.7 | 74.7 - 79.7 | 79.8 - 85.7 | 85.7 - 116.8 |  |  |  |
| n cases (T2D)/person-years | 156/34,148 | 139/35,204 | 107/36,752 | 123/39,193 | 135/45,700 |  |  |  |
| adj. for age and sex^1^ | 1 | 0.72 (0.57, 0.91) | 0.60 (0.47, 0.76) | 0.70 (0.56, 0.88) | 0.61 (0.48, 0.77) |  |  |  |
| + SES/ behaviours^2^ | 1 | 0.74 (0.59, 0.94) | 0.63 (0.49, 0.81) | 0.75 (0.59, 0.95) | 0.68 (0.53, 0.86) |  |  |  |
| + BMI^3^ | 1 | 0.74 (0.58, 0.93) | 0.63 (0.49, 0.80) | 0.75 (0.59, 0.95) | 0.69 (0.54, 0.88) |  |  |  |
| + prevalent CVD or cancer^4^ | 1 | 0.74 (0.59, 0.93) | 0.63 (0.49, 0.80) | 0.75 (0.59, 0.95) | 0.69 (0.54, 0.88) | 0.009 | 0.88 (0.82, 0.96) |  |
| **25-29.9** |  |  |  |  |  |  |  |  |
| n | 2,223 | 2,234 | 2,235 | 2,210 | 1,984 |  |  |  |
| PHD score, range | 36.5 - 68.4 | 68.4 - 74.7 | 74.7 - 79.8 | 79.8 - 85.7 | 85.7 - 117.8 |  |  |  |
| n cases (T2D)/person-years | 472/41,560 | 359/42,238 | 345/42,939 | 312/42,969 | 270/39,449 |  |  |  |
| adj. for age and sex^1^ | 1 | 0.84 (0.73, 0.96) | 0.74 (0.64, 0.85) | 0.65 (0.56, 0.75) | 0.65 (0.55, 0.75) |  |  |  |
| + SES/ behaviours^2^ | 1 | 0.85 (0.74, 0.97) | 0.78 (0.68, 0.89) | 0.69 (0.59, 0.80) | 0.69 (0.59, 0.81) |  |  |  |
| + BMI^3^ | 1 | 0.85 (0.75, 0.98) | 0.79 (0.68, 0.91) | 0.69 (0.59, 0.80) | 0.72 (0.62, 0.84) |  |  |  |
| + prevalent CVD or cancer^4^ | 1 | 0.85 (0.74, 0.97) | 0.78 (0.68, 0.90) | 0.68 (0.59, 0.79) | 0.71 (0.61, 0.83) | <0.0001 | 0.88 (0.83, 0.92) |  |
| **>30** |  |  |  |  |  |  |  |  |
| n | 783 | 772 | 746 | 643 | 581 |  |  |  |
| PHD score, range | 43.6 - 68.4 | 68.4 - 74.7 | 74.7 - 79.8 | 79.8 - 85.7 | 85.7 - 112.2 |  |  |  |
| n cases (T2D)/person-years | 290/12,810 | 257/12,903 | 218/13,017 | 158/11,566 | 155/10,639 |  |  |  |
| adj. for age and sex^1^ | 1 | 0.90 (0.76, 1.06) | 0.72 (0.61, 0.86) | 0.61 (0.50, 0.74) | 0.66 (0.54, 0.81) |  |  |  |
| + SES/ behaviours^2^ | 1 | 0.92 (0.78, 1.08) | 0.74 (0.62, 0.89) | 0.63 (0.52, 0.77) | 0.67 (0.55, 0.82) |  |  |  |
| + BMI^3^ | 1 | 0.90 (0.76, 1.06) | 0.74 (0.62, 0.88) | 0.62 (0.51, 0.76) | 0.66 (0.54, 0.80) |  |  |  |
| + prevalent CVD or cancer^4^ | 1 | 0.90 (0.76, 1.07) | 0.74 (0.62, 0.88) | 0.63 (0.51, 0.76) | 0.65 (0.53, 0.80) | <0.0001 | 0.83 (0.78, 0.89) | 0.08 |
| **Family history of diabetes** |  |  |  |  |  |  |  |  |
| **Family history** |  |  |  |  |  |  |  |  |
| n | 565 | 592 | 592 | 615 | 591 |  |  |  |
| PHD score, range | 46.0 - 68.4 | 68.4 - 74.7 | 74.7 - 79.7 | 79.8 - 85.7 | 85.7 - 114.7 |  |  |  |
| n cases (T2D)/person-years | 159/10,055 | 148/11,179 | 144/11,159 | 122/11,830 | 121/11,703 |  |  |  |
| adj. for age and sex^1^ | 1 | 0.92 (0.74, 1.14) | 0.83 (0.66, 1.04) | 0.73 (0.58, 0.92) | 0.66 (0.52, 0.85) |  |  |  |
| + SES/ behaviours^2^ | 1 | 0.93 (0.75, 1.16) | 0.84 (0.67, 1.06) | 0.74 (0.58, 0.94) | 0.68 (0.53, 0.88) |  |  |  |
| + BMI^3^ | 1 | 0.96 (0.77, 1.20) | 0.85 (0.68, 1.08) | 0.80 (0.63, 1.02) | 0.75 (0.58, 0.97) |  |  |  |
| + prevalent CVD or cancer^4^ | 1 | 0.97 (0.78, 1.21) | 0.84 (0.67, 1.07) | 0.80 (0.63, 1.02) | 0.75 (0.58, 0.96) | 0.008 | 0.89 (0.82, 0.96) |  |
| **No family history** |  |  |  |  |  |  |  |  |
| n | 4,180 | 4,153 | 4,150 | 4,130 | 4,154 |  |  |  |
| PHD score, range | 33.9 - 68.4 | 68.4 - 74.7 | 74.7 - 79.8 | 79.8 - 85.7 | 85.7 - 117.8 |  |  |  |
| n cases (T2D)/person-years | 759/78,463 | 607/79,166 | 526/81,549 | 471/81,898 | 439/84,086 |  |  |  |
| adj. for age and sex^1^ | 1 | 0.81 (0.73, 0.90) | 0.67 (0.60, 0.75) | 0.60 (0.53, 0.67) | 0.57 (0.51, 0.65) |  |  |  |
| + SES/ behaviours^2^ | 1 | 0.84 (0.75, 0.93) | 0.72 (0.64, 0.80) | 0.64 (0.57, 0.73) | 0.63 (0.56, 0.72) |  |  |  |
| + BMI^3^ | 1 | 0.82 (0.74, 0.91) | 0.72 (0.64, 0.80) | 0.65 (0.58, 0.73) | 0.67 (0.59, 0.76) |  |  |  |
| + prevalent CVD or cancer^4^ | 1 | 0.82 (0.74, 0.91) | 0.72 (0.64, 0.80) | 0.65 (0.58, 0.73) | 0.66 (0.59, 0.75) | <0.0001 | 0.85 (0.82, 0.89) | 0.17 |

Quintile 1 (Q1) represents the lowest adherence to the PHD, while Quintile 5 (Q5) represents the highest adherence. The total possible PHD score ranges from 0 (lowest) to 140 (highest). Data are HR (95% CI). In the continuous analyses, the HRs and 95% CIs were estimated per 10-unit higher score of the PHD score. ^1^Model 1: adjusted for age (years) and sex (male or female). ^2^Model 2: adjusted for factors in model 1 plus physical activity (inactive, moderately inactive, moderately active, or active), smoking status (never, former, or current), level of education (none, O-level, A-level, or degree), use of vitamin supplements (yes/no), family history of diabetes (yes/no), alcohol intake (g/d) and energy intake (continuous, kcal/day). ^3^Model 3: adjusted for factors in Model 2 plus body mass index (kg/m^2^). ^4^Model 4: adjusted for factors in Model 3 plus prevalent CVD or cancer. P-interaction was obtained by including comparing a model without and a model with cross-product terms of the PHD score (continuous) and each of the covariates using a likelihood ratio test. BMI= body mass index, CVD= cardiovascular disease, HR= hazard ratio, PHD= planetary health diet, SES= socioeconomic status, T2D= type 2 diabetes.
