# Supplementary material for: The association of the planetary health diet with type 2 diabetes incidence and greenhouse gas emissions: Findings from the EPIC-Norfolk prospective cohort study
Source: PLoS Med. 2025 Sep 16;22(9):e1004633. doi: 10.1371/journal.pmed.1004633 (PMC12440362; doi:10.1371/journal.pmed.1004633)
Supplement: S3 Table — Quintile 1 (Q1) represents the lowest adherence to the PHD, while Quintile 5 (Q5) represents the highest adherence. The total possible PHD score ranges from 0 (lowest) to 140 (highest). Data are HR (95% CI). In the continuous analyses, the HRs and 95% CIs were estimated per 10-point higher score of the PHD score. 1Model 1: adjusted for age (years) and sex (male or female). 2Model 2: adjusted for factors in model 1 plus physical activity (inactive, moderately inactive, moderately active, or active), smoking status (never, former, or current), level of education (primary/none, O-level, A-level, or degree), use of vitamin supplements (yes/no), family history of diabetes (yes/no), alcohol intake (g/d) and energy intake (continuous, kcal/d). 3Model 3: adjusted for factors in Model 2 plus body mass index (kg/m2). 4Model 4: adjusted for factors in Model 3 plus prevalent CVD or cancer. 5Model 5: adjusted for factors in Model 4 plus use of hormone replacement therapy at baseline (females only). 6Model 5: adjusted for factors in Model 4 plus the Mediterranean diet score. 7Model 5: adjusted for factors in Model 4 plus cardiometabolic risk markers, i.e., waist circumference, systolic and diastolic blood pressure, HbA1c, plasma triglycerides, total cholesterol, low-density lipoprotein cholesterol, high-density lipoprotein cholesterol, and C-reactive protein. *HR (95% CI) per unit higher score. BMI, body mass index; CRM, cardiometabolic risk markers; CVD, cardiovascular disease; FFQ, food frequency questionnaire; HbA1c, glycated haemoglobin; HR, hazard ratio; PHD, planetary health diet; SES, socioeconomic status; T2D, type 2 diabetes. (DOCX) [file pmed.1004633.s008.docx]

| **S3 Table. Association between the Planetary Health Diet and risk of type 2 diabetes in sensitivity analyses** | | | | | | | |
| --- | --- | --- | --- | --- | --- | --- | --- |
|  | **Q1** | **Q2** | **Q3** | **Q4** | **Q5** | **p-trend** | **HR (95% CI) per**  **10-points** |
| Excluding baseline Hba1c >6.5 or T2D cases within 2y of follow-up | | | | | | | |
| n | 1,970 | 1,925 | 2,004 | 2,024 | 2,126 |  |  |
| range | 33.9 - 68.4 | 68.4 - 74.7 | 74.7 - 79.8 | 79.8 - 85.7 | 85.7 - 117.8 |  |  |
| cases/person-years | 317/37,451 | 249/36,921 | 238/39,358 | 201/40,090 | 207/42,527 |  |  |
| adj. for age and sex^1^ | 1 | 0.83 (0.71, 0.98) | 0.68 (0.57, 0.80) | 0.60 (0.50, 0.71) | 0.57 (0.48, 0.68) |  |  |
| + SES/ behaviours^2^ | 1 | 0.84 (0.72, 0.99) | 0.70 (0.59, 0.83) | 0.64 (0.53, 0.76) | 0.62 (0.52, 0.75) |  |  |
| + BMI^3^ | 1 | 0.86 (0.73, 1.01) | 0.67 (0.56, 0.79) | 0.65 (0.55, 0.78) | 0.66 (0.55, 0.79) |  |  |
| + prevalent CVD or cancer^4^ | 1 | 0.86 (0.73, 1.01) | 0.66 (0.56, 0.79) | 0.65 (0.55, 0.78) | 0.65 (0.54, 0.79) | <0.0001 | 0.85 (0.80, 0.90) |
| Excluding those with prevalent CVD or cancer | | | | | | | |
| n | 4,312 | 4,300 | 4,294 | 4,304 | 4,308 |  |  |
| range | 33.9 - 68.4 | 68.4 - 74.7 | 74.7 - 79.8 | 79.8 - 85.7 | 85.7 - 117.8 |  |  |
| cases/person-years | 837/82,420 | 687/83,841 | 606/85,622 | 533/86,612 | 491/88,475 |  |  |
| adj. for age and sex^1^ | 1 | 0.84 (0.76, 0.92) | 0.70 (0.63, 0.78) | 0.63 (0.57, 0.71) | 0.57 (0.51, 0.64) |  |  |
| + SES/ behaviours^2^ | 1 | 0.85 (0.77, 0.94) | 0.74 (0.66, 0.82) | 0.66 (0.59, 0.74) | 0.61 (0.54, 0.69) |  |  |
| + BMI^3^ | 1 | 0.84 (0.76, 0.93) | 0.73 (0.66, 0.82) | 0.68 (0.61, 0.76) | 0.65 (0.58, 0.73) | <0.0001 | 0.85 (0.80, 0.88) |
| Excluding those with extremely low or high energy intakes | | | | | | | |
| N | 4,680 | 4,686 | 4,675 | 4,679 | 4,681 |  |  |
| range | 33.9 - 68.4 | 68.4 - 74.7 | 74.7 - 79.8 | 79.8 - 85.7 | 85.7 - 117.8 |  |  |
| cases/person-years | 904/87,273 | 748/89,100 | 660/91,361 | 582/92,474 | 552/94,486 |  |  |
| adj. for age and sex^1^ | 1 | 0.84 (0.76, 0.92) | 0.70 (0.64, 0.78) | 0.62 (0.56, 0.69) | 0.59 (0.53, 0.66) |  |  |
| + SES/ behaviours^2^ | 1 | 0.85 (0.78, 0.94) | 0.74 (0.67, 0.82) | 0.66 (0.59, 0.74) | 0.64 (0.57, 0.71) |  |  |
| + BMI^3^ | 1 | 0.84 (0.77, 0.93) | 0.73 (0.66, 0.81) | 0.68 (0.61, 0.75) | 0.68 (0.61, 0.76) |  |  |
| + prevalent CVD or cancer^4^ | 1 | 0.85 (0.77, 0.93) | 0.73 (0.66, 0.81) | 0.68 (0.61, 0.75) | 0.68 (0.61, 0.76) | <0.0001 | 0.86 (0.83, 0.89) |
| Adjustment for use of hormone replacement therapy (women only) | | | | | | | |
| n | 1,883 | 2,475 | 2,635 | 2,910 | 3,152 |  |  |
| range | 41.7 - 68.4 | 68.4 - 74.7 | 74.7 - 79.8 | 79.8 - 85.7 | 85.7 - 117.8 |  |  |
| cases/person-years | 322/36,836 | 357/49,097 | 342/53,761 | 331/59,468 | 354/65,109 |  |  |
| adj. for age and sex^1^ | 1 | 0.80 (0.69, 0.93) | 0.70 (0.61, 0.82) | 0.62 (0.53, 0.72) | 0.59 (0.51, 0.68) |  |  |
| + SES/ behaviours^2^ | 1 | 0.83 (0.71, 0.96) | 0.73 (0.63, 0.85) | 0.67 (0.57, 0.78) | 0.64 (0.55, 0.75) |  |  |
| + BMI^3^ | 1 | 0.82 (0.71, 0.95) | 0.75 (0.65, 0.87) | 0.70 (0.60, 0.82) | 0.71 (0.61, 0.82) |  |  |
| + prevalent CVD or cancer^4^ | 1 | 0.83 (0.71, 0.96) | 0.76 (0.65, 0.88) | 0.70 (0.60, 0.82) | 0.71 (0.61, 0.83) |  |  |
| + HRT use^5^ | 1 | 0.83 (0.71, 0.96) | 0.75 (0.65, 0.88) | 0.70 (0.60, 0.82) | 0.71 (0.61, 0.82) | <0.0001 | 0.88 (0.84, 0.93) |
| Adjustment for the Mediterranean diet | | | | | | | |
| n | 4,745 | 4,745 | 4,742 | 4,745 | 4,745 |  |  |
| range | 33.9 - 68.4 | 68.4 - 74.7 | 74.7 - 79.8 | 79.8 - 85.7 | 85.7 - 117.8 |  |  |
| cases/person-years | 918/88,517 | 755/90,344 | 670/92,708 | 593/93,728 | 560/95,788 |  |  |
| adj. for age and sex^1^ | 1 | 0.84 (0.76, 0.92) | 0.70 (0.64, 0.78) | 0.63 (0.56, 0.70) | 0.59 (0.53, 0.66) |  |  |
| + SES/ behaviours^2^ | 1 | 0.86 (0.78, 0.94) | 0.74 (0.67, 0.82) | 0.66 (0.60, 0.74) | 0.64 (0.57, 0.71) |  |  |
| + BMI^3^ | 1 | 0.85 (0.77, 0.93) | 0.74 (0.67, 0.81) | 0.68 (0.61, 0.76) | 0.68 (0.61, 0.76) |  |  |
| + prevalent CVD or cancer^4^ | 1 | 0.85 (0.77, 0.94) | 0.74 (0.67, 0.81) | 0.68 (0.61, 0.76) | 0.68 (0.61, 0.76) |  |  |
| + Mediterranean diet score^6^ | 1 | 0.83 (0.75, 0.92) | 0.72 (0.65, 0.81) | 0.67 (0.59, 0.75) | 0.67 (0.58, 0.77) | <0.0001 | 0.85 (0.81, 0.89) |
| Adjustment for cardiometabolic risk markers (CRM) | | | | | | | |
| n | 1,342 | 1,326 | 1,413 | 1,404 | 1,482 |  |  |
| range | 41.2 - 68.4 | 68.4 - 74.7 | 74.7 - 79.8 | 79.8 - 85.7 | 85.7 - 117.8 |  |  |
| cases/person-years | 242/25,365 | 181/25,440 | 183/27,596 | 164/27,664 | 156/29,793 |  |  |
| adj. for age and sex^1^ | 1 | 0.74 (0.64, 0.85) | 0.68 (0.59, 0.79) | 0.63 (0.54, 0.73) | 0.58 (0.49, 0.68) |  |  |
| + SES/ behaviours^2^ | 1 | 0.75 (0.65, 0.87) | 0.71 (0.61, 0.82) | 0.67 (0.57, 0.78) | 0.63 (0.53, 0.74) |  |  |
| + BMI^3^ | 1 | 0.74 (0.64, 0.86) | 0.68 (0.59, 0.79) | 0.68 (0.58, 0.79) | 0.66 (0.56, 0.77) |  |  |
| + prevalent CVD or cancer^4^ | 1 | 0.75 (0.64, 0.86) | 0.68 (0.59, 0.79) | 0.68 (0.58, 0.79) | 0.66 (0.56, 0.77) |  |  |
| + CRM^7^ | 1 | 0.82 (0.71, 0.94) | 0.76 (0.65, 0.88) | 0.62 (0.53, 0.73) | 0.75 (0.64, 0.88) | <0.0001 | 0.88 (0.83, 0.93) |
| Using only baseline FFQ | | | | | | | |
| n | 4,745 | 4,745 | 4,742 | 4,745 | 4,745 |  |  |
| range | 33.9 - 68.4 | 68.4 - 74.7 | 74.7 - 79.8 | 79.8 - 85.7 | 85.7 - 117.8 |  |  |
| cases/person-years | 918/88,517 | 755/90,344 | 670/92,708 | 593/93,728 | 560/95,788 |  |  |
| adj. for age and sex^1^ | 1 | 0.81 (0.73, 0.89) | 0.69 (0.63, 0.77) | 0.61 (0.55, 0.68) | 0.58 (0.52, 0.64) |  |  |
| + SES/ behaviours^2^ | 1 | 0.83 (0.76, 0.92) | 0.74 (0.67, 0.82) | 0.66 (0.59, 0.73) | 0.64 (0.57, 0.71) |  |  |
| + BMI^3^ | 1 | 0.84 (0.76, 0.92) | 0.74 (0.67, 0.82) | 0.68 (0.61, 0.75) | 0.68 (0.61, 0.76) |  |  |
| + prevalent CVD or cancer^4^ | 1 | 0.84 (0.76, 0.92) | 0.74 (0.67, 0.82) | 0.68 (0.61, 0.75) | 0.67 (0.60, 0.75) | <0.0001 | 0.86 (0.83, 0.89) |
| Evaluating adherence using a dichotomous approach | | | | | | | |
| n | 0.0 - 2.5 | 3.0 - 3.0 | 3.5 - 4.0 | 4.5 - 5.0 | 5.5 - 10.5 |  |  |
| range | 4,871 | 5,322 | 6,272 | 4,207 | 3,050 |  |  |
| cases/person-years | 829/93,939 | 829/102,340 | 920/121,963 | 556/82,472 | 362/60,372 |  |  |
| adj. for age and sex^1^ | 1 | 0.93 (0.82, 1.04) | 0.87 (0.78, 0.98) | 0.80 (0.69, 0.92) | 0.79 (0.68, 0.92) |  |  |
| + SES/ behaviours^2^ | 1 | 0.93 (0.83, 1.05) | 0.89 (0.79, 1.00) | 0.83 (0.72, 0.96) | 0.82 (0.70, 0.96) |  |  |
| + BMI^3^ | 1 | 0.96 (0.85, 1.08) | 0.87 (0.77, 0.98) | 0.84 (0.73, 0.97) | 0.84 (0.72, 0.99) |  |  |
| + prevalent CVD or cancer^4^ | 1 | 0.95 (0.84, 1.07) | 0.86 (0.76, 0.97) | 0.84 (0.73, 0.97) | 0.84 (0.71, 0.98) | 0.002 | 0.95 (0.93, 0.98)* |

Data are HR (95% CI). In the continuous analyses, the HRs and 95% CIs were estimated per 10-unit higher score of the PHD score. ^1^Model 1: adjusted for age (years) and sex (male or female). ^2^Model 2: adjusted for factors in model 1 plus physical activity (inactive, moderately inactive, moderately active, or active), smoking status (never, former, or current), level of education (none, O-level, A-level, or degree), use of vitamin supplements (yes/no), family history of diabetes (yes/no), alcohol intake (g/d) and energy intake (continuous, kcal/day). ^3^Model 3: adjusted for factors in Model 2 plus body mass index (kg/m^2^). ^4^Model 4: adjusted for factors in Model 3 plus prevalent CVD or cancer. ^5^Model 5: adjusted for factors in Model 4 plus use of hormone replacement therapy at baseline (women only). ^6^Model 5: adjusted for factors in Model 4 plus the Mediterranean diet score. ^7^Model 5: adjusted for factors in Model 4 plus cardiometabolic risk markers i.e., waist circumference, systolic and diastolic blood pressure, HbA1c, plasma triglycerides, total cholesterol, low-density lipoprotein cholesterol, high-density lipoprotein cholesterol, and C-reactive protein. *HR (95% CI) per unit higher score. BMI= body mass index, CRM= cardiometabolic risk markers, CVD= cardiovascular disease, FFQ= food frequency questionnaire, HbA1c= glycated haemoglobin, HR= hazard ratio PHD= planetary health diet, SES= socioeconomic status, T2D= type 2 diabetes.
