# Supplementary material for: The association of the planetary health diet with type 2 diabetes incidence and greenhouse gas emissions: Findings from the EPIC-Norfolk prospective cohort study
Source: PLoS Med. 2025 Sep 16;22(9):e1004633. doi: 10.1371/journal.pmed.1004633 (PMC12440362; doi:10.1371/journal.pmed.1004633)
Supplement: S4 Table — 1For each nutritional biomarker, the analyses were performed for a subsample of participants who had data for the biomarker, hence the sample sizes vary for each biomarker. All Cox models were adjusted for age (years), sex (male or female), physical activity (inactive, moderately inactive, moderately active, active), energy intake (kcal/d), smoking status (never, former, or current), level of education (primary/none, O-level, A-level, or degree), use of vitamin supplements (yes/no), family history of diabetes (yes/no), alcohol intake (g/d), and BMI (kg/m2), and prevalent CVD or cancer. 2HR (95% CI) of the association between the PHD and risk of T2D; HRs (95% CIs) estimate the risk of type 2 diabetes per 10-points higher PHD score. 3HR (95% CI) of the association between the nutritional biomarker and risk of T2D, in a model that additionally adjusted for the PHD; the HR (95% CI) estimate the risk of type 2 diabetes per-SD of nutritional biomarker. 4HR (95%) CI of the association between the PHD and risk of T2D, in a model adjusted for the nutritional biomarker. Proportion of the PHD-T2D association explained by the nutritional biomarker. BMI, body mass index; PHD, planetary health diet; HR, hazard ratio; SD, standard deviation; T2D, type 2 diabetes. (DOCX) [file pmed.1004633.s009.docx]

| **S4 Table. Evaluation of the potential effect of nutritional biomarkers on the association between the Planetary Health Diet and risk of type 2 diabetes^1^** | | | | | | | |
| --- | --- | --- | --- | --- | --- | --- | --- |
|  | **n** | **cases** | **Person-**  **years** | **HR (95% CI)**  **(PHD and T2D)^2^** | **HR (95% CI)**  **(Biomarker and**  **T2D – adjusting**  **for PHD)^3^** | **HR (95% CI)**  **(PHD and**  **T2D – adjusting**  **for biomarker^4^** | **Proportion (%)**  **explained**  **(95% CI)^5^** |
| Vitamin C | 20,887 | 3,028 | 407,664 | 0.85 (0.82, 0.88) | 0.80 (0.77, 0.83) | 0.87 (0.84, 0.91) | 14.8 (10.5, 21.3) |
| Lycopene | 6,800 | 1,101 | 115,607 | 0.87 (0.82, 0.92) | 0.95 (0.89, 1.02) | 0.87 (0.82, 0.93) | 3.9 (-1.3, 11.7) |
| Cryptoxanthin | 6,801 | 1,101 | 115,620 | 0.87 (0.82, 0.92) | 0.89 (0.83, 0.96) | 0.88 (0.82, 0.93) | 6.2 (2.2, 14.6) |
| α-carotene | 6,799 | 1,101 | 115,571 | 0.87 (0.82, 0.92) | 0.84 (0.78, 0.90) | 0.89 (0.84, 0.95) | 16.8 (8.5, 36.4) |
| β-carotene | 6,801 | 1,101 | 115,620 | 0.87 (0.82, 0.92) | 0.78 (0.72, 0.84) | 0.89 (0.84, 0.95) | 18.4 (10.2, 38.7) |
| Zeaxanthin | 6,797 | 1,100 | 115,549 | 0.87 (0.81, 0.92) | 0.89 (0.84, 0.95) | 0.87 (0.82, 0.92) | 1.7 (-0.6, 5.6) |
| Lutein | 6,801 | 1,101 | 115,620 | 0.87 (0.82, 0.92) | 0.86 (0.80, 0.92) | 0.88 (0.83, 0.94) | 10.4 (4.8, 22.7) |
| α-tocopherol | 6,801 | 1,101 | 115,620 | 0.87 (0.82, 0.92) | 1.18 (1.12, 1.25) | 0.86 (0.81, 0.91) | -7.0 (-16.3, -2.0) |
| γ-tocopherol | 6,793 | 1,099 | 115,492 | 0.87 (0.81, 0.92) | 1.24 (1.17, 1.32) | 0.87 (0.82, 0.93) | 4.9 (-0.2, 12.8) |

^1^For each nutritional biomarker, the analyses were performed for a subsample of participants who had data for the biomarker, hence the sample sizes vary for each biomarker. All Cox models were adjusted for age (years), sex (male or female), physical activity (inactive, moderately inactive, moderately active, active), energy intake (kcal/day), smoking status (never, former, or current), level of education (none, O-level, A-level, or degree), use of vitamin supplements (yes/no), family history of diabetes (yes/no), alcohol intake (g/d), and BMI (kg/m^2^), and prevalent CVD or cancer. ^2^HR (95% CI) of the association between the PHD and risk of T2D; HRs (95% CIs) estimate the risk of type 2 diabetes per 10-points higher PHD score. ^3^HR (95% CI) of the association between the nutritional biomarker and risk of T2D, in a model that additionally adjusted for the PHD; the HR (95% CI) estimate the risk of type 2 diabetes per-SD of nutritional biomarker. ^4^HR (95%) CI of the association between the PHD and risk of T2D, in a model adjusted for the nutritional biomarker. Proportion of the PHD-T2D association explained by the nutritional biomarker. BMI=body mass index, PHD=planetary health diet, HR= hazard ratio, SD= standard deviation, T2D= type 2 diabetes.
