# Supplementary figures and images for: The association of the planetary health diet with type 2 diabetes incidence and greenhouse gas emissions: Findings from the EPIC-Norfolk prospective cohort study
Source: PLoS Med. 2025 Sep 16;22(9):e1004633. doi: 10.1371/journal.pmed.1004633 (PMC12440362; doi:10.1371/journal.pmed.1004633)

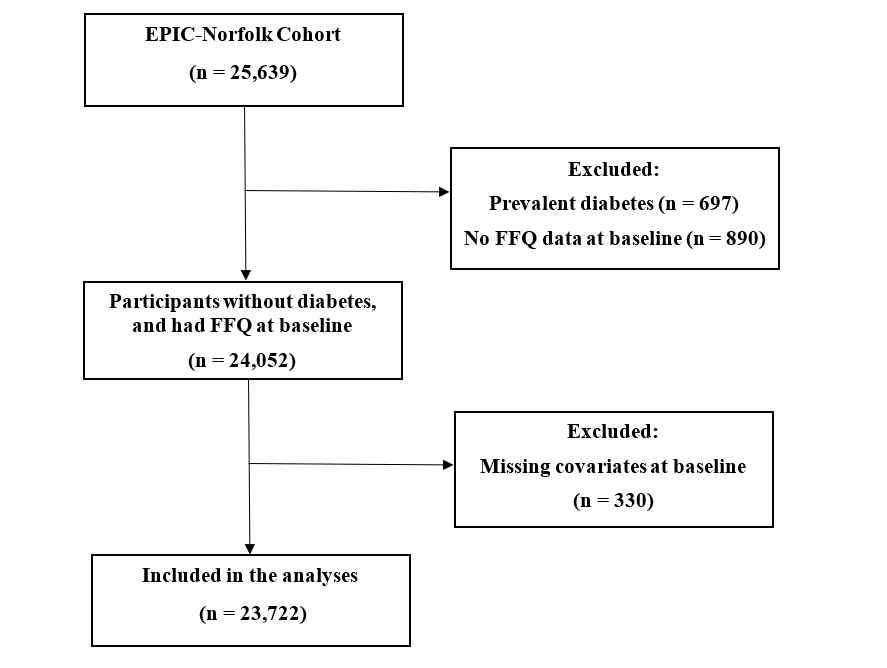

Supplement: S1 Fig — FFQ, food frequency questionnaire. (TIF) [file pmed.1004633.s010.tif]

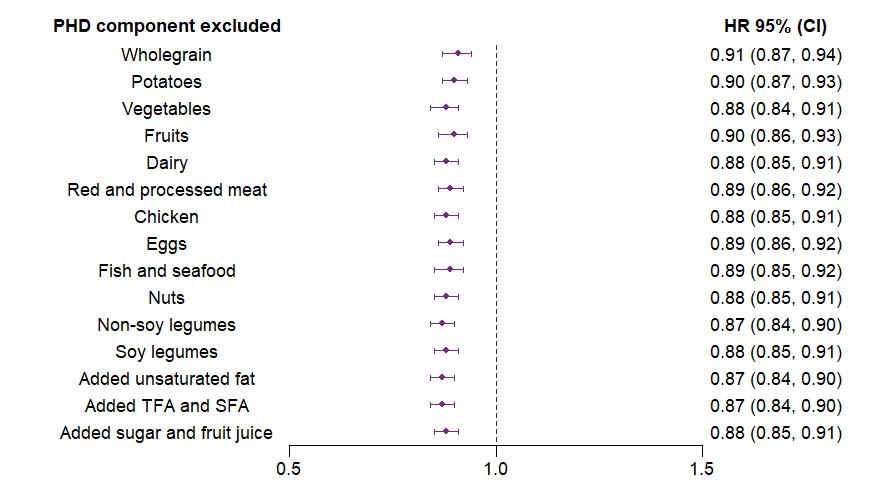

Supplement: S2 Fig — Data are HRs and 95% CIs per 10-point higher PHD score obtained by fitting the most adjusted Cox model. The model adjusted for age (years), sex (male or female), plus physical activity (inactive, moderately inactive, moderately active, or active), smoking status (never, former, or current), level of education (primary/none, O-level, A-level, or degree), use of vitamin supplements (yes/no), family history of diabetes (yes/no), alcohol intake (g/d), energy intake (continuous, kcal/d), body mass index (kg/m2), prevalent CVD or cancer, and the PHD component excluded from the calculation of the PHD score. BMI, body mass index; CVD, cardiovascular disease; HR, hazard ratio; PHD, planetary health diet. (TIF) [file pmed.1004633.s011.tif]

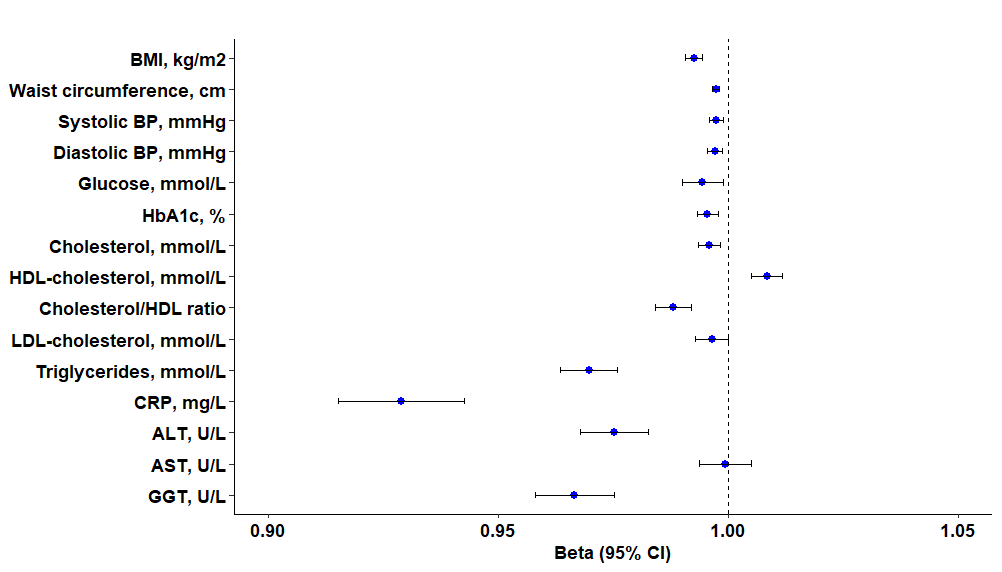

Supplement: S3 Fig — The cardiometabolic risk factors were log-transformed prior to the analysis, and the beta estimates were back-transformed before being plotted. Thus, a null effect corresponds to an estimate of 1.00 (dashed vertical line). The models were adjusted for age (years), sex(male or female), physical activity (inactive, moderately inactive, moderately active, or active), smoking status (never, former, or current), level of education (primary/none, O-level, A-level, or degree), use of multivitamin supplements (yes/no), family history of diabetes (yes/no), alcohol intake (g/d), energy intake (kcal/d), and BMI (kg/m2) (except when BMI was the outcome). ALT, Alanine aminotransferase; AST, Aspartate aminotransferase; BMI, body mass index; CRP, C-reactive protein; GGT, Gamma-glutamyltransferase; HbA1c, glycated haemoglobin; HDL, high density lipoprotein; LDL, low density lipoprotein. (TIF) [file pmed.1004633.s012.tif]

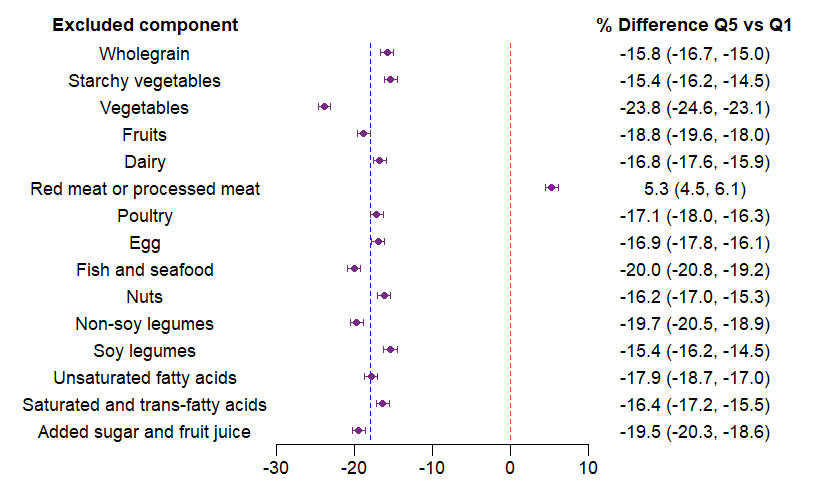

Supplement: S4 Fig — The PHD score was modelled as quintiles, and the models were adjusted for age (years), sex (male or female), alcohol intake (g/d), energy intake (kcal/d), and the score for the PHD food component excluded from the derivation of the score. The models included a random intercept for participant identifier to account to the repeated measurements of the PHD score and greenhouse gas (GHG) emissions. The estimates represent the expected percentage differences (95% CI) in the GHG emissions (kgCO2eq/d) for participants in quintile 5 compared to those in quintile 1 (reference category). The red vertical line is the reference line for no association between the PHD score and GHG emissions. The blue vertical line is positioned at the value of the point estimate obtained from the linear regression analysis for the association between the PHD score (based on all components) and GHG emissions, adjusting for age, sex, alcohol intake, and energy intake. GHG, greenhouse gas; PHD, planetary health diet; Q1and Q5, quintiles 1 and 5, respectively. (TIF) [file pmed.1004633.s013.tif]
